# Discovery, structure and mechanism of a tetraether lipid synthase

**DOI:** 10.1038/s41586-022-05120-2

**Published:** 2022-07-26

**Authors:** Cody T. Lloyd, David F. Iwig, Bo Wang, Matteo Cossu, William W. Metcalf, Amie K. Boal, Squire J. Booker

**Affiliations:** 1 Department of Biochemistry and Molecular Biology, Pennsylvania State University, University Park, PA USA; 2 The Howard Hughes Medical Institute, Pennsylvania State University, University Park, PA USA; 3 Department of Chemistry, Pennsylvania State University, University Park, PA USA; 4 Department of Microbiology, University of Illinois Urbana–Champaign, Urbana, IL USA; 5 Institute for Genomic Biology, University of Illinois Urbana–Champaign, Urbana, IL USA

**Keywords:** Metalloproteins, X-ray crystallography

## Abstract

Archaea synthesize isoprenoid-based ether-linked membrane lipids, which enable them to withstand extreme environmental conditions, such as high temperatures, high salinity, and low or high pH values^1–5^. In some archaea, such as *Methanocaldococcus jannaschii*, these lipids are further modified by forming carbon–carbon bonds between the termini of two lipid tails within one glycerophospholipid to generate the macrocyclic archaeol or forming two carbon–carbon bonds between the termini of two lipid tails from two glycerophospholipids to generate the macrocycle glycerol dibiphytanyl glycerol tetraether (GDGT)^1,2^. GDGT contains two 40-carbon lipid chains (biphytanyl chains) that span both leaflets of the membrane, providing enhanced stability to extreme conditions. How these specialized lipids are formed has puzzled scientists for decades. The reaction necessitates the coupling of two completely inert sp^3^-hybridized carbon centres, which, to our knowledge, has not been observed in nature. Here we show that the gene product of *mj0619* from *M. jannaschii*, which encodes a radical *S*-adenosylmethionine enzyme, is responsible for biphytanyl chain formation during synthesis of both the macrocyclic archaeol and GDGT membrane lipids^6^. Structures of the enzyme show the presence of four metallocofactors: three [Fe_4_S_4_] clusters and one mononuclear rubredoxin-like iron ion. In vitro mechanistic studies show that Csp^3^–Csp^3^ bond formation takes place on fully saturated archaeal lipid substrates and involves an intermediate bond between the substrate carbon and a sulfur of one of the [Fe_4_S_4_] clusters. Our results not only establish the biosynthetic route for tetraether formation but also improve the use of GDGT in GDGT-based paleoclimatology indices^7–10^.

## Abstract (web summary)

In *Methanocaldococcus jannaschii*, a radical *S*-adenosylmethionine enzyme catalyses the formation of the biphytanyl chain.

## Main

**Fig. 1:**
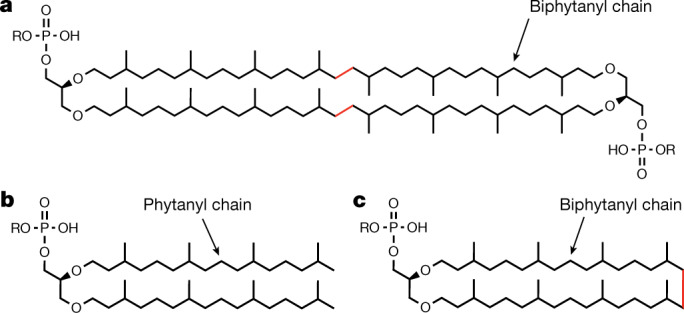
Structures of saturated isoprenoid-based archaeal lipids. **a**–**c**, Structures of a GDGT (**a**), diether archaeal lipid (archaeol when R = H and archaetidylglycerol when R = glycerol) (**b**) and macrocyclic archaeol (**c**). Archaeols contain two 20-carbon chains called phytanyl chains, whereas archaeol lipid macrocycles contain one (observed in macrocyclic archaeols) or two (observed in GDGTs) 40-carbon chains called biphytanyl chains. The red C–C bond shown in the archaeol diether macrocycle (**b**) and GDGT (**c**) is formed during biphytanyl chain synthesis. All carbon chains are appended to an *sn*-glycero-1-phophate backbone.

GDGT is a unique membrane-spanning macrocyclic ether lipid found predominantly in archaea^1,2^ (Fig. 1a). The rigid structure of GDGT imparts membrane stability, enabling organisms that contain it to thrive under extreme environmental conditions (for example, high temperatures, high salt concentrations, and low or high pH values)^3–5^. Unlike eukaryotic and bacterial membrane lipids, which are synthesized as straight-chain fatty acids, archaeal lipids are synthesized from isopentenyl diphosphate and dimethylallyl diphosphate isoprenoid building blocks to form saturated branched carbon chains known as phytanyl. These chains are appended to an *sn*-glycero-1-phosphate backbone via an ether bond^1,2,4^ (Fig. 1a–c). In archaeal extremophiles, such as *M. jannaschii*, the phytanyl chain is modified by the formation of a C–C bond that tethers the lipid tails together to form the 40-carbon biphytanyl chain, which is observed in both macrocyclic archaeol (Fig. 1b) and GDGT^2,6^. Moreover, several studies have shown that environmental factors, such as temperature, influence the synthesis of the biphytanyl chain^11–13^. Thus, GDGT is an ideal ecological proxy used to reconstruct geological temperature changes^7–10^. However, the gene responsible for biphytanyl chain formation was as yet unknown, consequently limiting the efficacy of GDGT as a biomarker because it necessitated that GDGT-producing organisms be identified experimentally. It must be mentioned that after the submission of this work, a paper by Zeng et al. was published that identified a GDGT synthase from *Sulfolobus acidocaldarius* through in vivo complementation studies, which they named tetraether synthase^14^.

**Extended Data Fig. 1:**
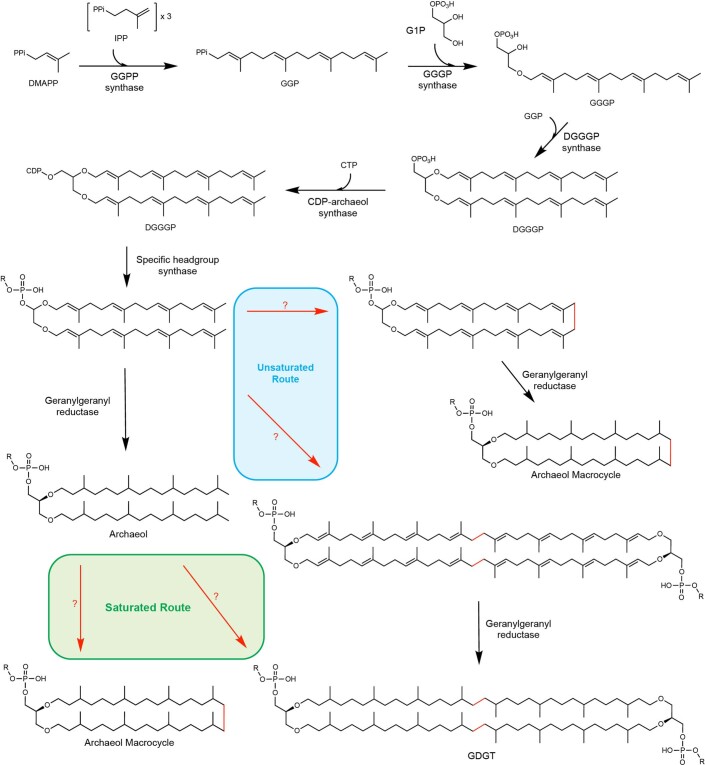
Saturated and unsaturated biosynthesis pathways for biphytanyl chain formation in macrocyclic Archaeol and GDGT. Archaeal membrane lipid biosynthesis is well established through formation of the fully saturated diether lipid Archaeol. However, how the biphytanyl chain is formed is unknown. Two potential pathways for generating the biphytanyl chain have been proposed: a pathway involving a saturated substrate and a pathway involving an unsaturated substrate, both of which entail the formation of a Csp^3^-Csp^3^ bond between the termini of two lipid chains. The distinction between the two routes, however, is when the biphytanyl chain is formed in relation to saturation of the lipid chain by geranylgeranyl reductase (GGR). In the unsaturated route, the biphytanyl chain is formed prior to chain saturation by GGR. Therefore, the substrate for the reaction is an unsaturated lipid: digeranylgeranyl glycerol phosphate (DGGGP) with R=H or a polar headgroup. In the saturated route, the fully saturated lipid is formed by GGR-mediated chain reduction prior to biphytanyl chain formation. Therefore, the substrate for the reaction contains fully saturated chains: archaeol when R=H and archaetidylglycerol when R=glycerol. Abbreviations: dimethylallyl diphosphate (DMAPP), isopentenyl pyrophosphate (IPP), geranylgeranyl pyrophosphate synthase (GGPP synthase), geranylgeranyl pyrophosphate (GGP), *sn*-glycerol-1-phosphate (G1P), geranylgeranyl glycerol phosphate (GGGP), digeranylgeranyl glycerol phosphate (DGGGP), cytidine diphosphate (CDP), glycerol dibiphytanyl glycerol tetraether (GDGT).

The sole unannotated step in archaeal lipid biosynthesis was the construction of the biphytanyl chain during the formation of GDGT and macrocyclic archaeol (Fig. 1b,c). The inability to characterize this reaction has led to a disagreement over the biosynthetic route to tether the chains together^15,16^ (Extended Data Fig. 1). Formation of the biphytanyl chain independent of the biosynthetic route would necessitate two sequential C–H activations on the terminal Csp^3^ carbons, which would require challenging radical chemistry. Given that all characterized GDGT structures are fully saturated, one hypothesis is that the biphytanyl chain is synthesized from saturated lipids (that is, saturated route). In this scenario, the biosynthesis of GDGT occurs after saturation of the geranylgeranyl chain by geranylgeranyl reductase (GGR). However, the formation of a C–C bond between two phytanyl chains would require the ability to store high-energy radical intermediates. Therefore, an opposing hypothesis is that the C–C bond forms before chain saturation, thus allowing for bond formation by radical addition into a π system (that is, unsaturated route). However, a clear precedent for either pathway has not been established.

The construction of archaeal membrane-spanning lipids from inert archaeal lipid substrates requires radical-based chemistry and the coupling of two terminal methyl carbons. Nature uses various strategies to initiate radical-based chemistry, such as the chemistry performed by enzymes in the radical *S-*adenosylmethionine (SAM) superfamily. Although many other enzymes can initiate radical-based chemistry, most of them require O_2_. However, this strategy would not be consistent with the metabolism of many of the organisms that produce GDGT, which are largely obligate anaerobes. Radical SAM (RS) enzymes cleave SAM reductively to yield methionine and a 5′-deoxyadenosyl 5′-radical (5′-dA•). The resulting 5′-dA• is a potent oxidant that usually initiates catalysis by abstracting a substrate hydrogen atom (H•), often from an unreactive carbon^17–19^. Thus, RS chemistry is a viable strategy for biphytanyl formation. Here, through the use of X-ray crystallography, mass spectrometry and in vitro activity determinations, we show that the RS enzyme encoded by the gene *mj0619* from *M. jannaschii*, which we designate GDGT–macrocyclic archaeol synthase (GDGT–MAS), catalyses the formation of the biphytanyl chain during archaeal diether macrocycle and GDGT biosynthesis. Moreover, in vitro catalysis was achieved with a fully saturated archaeal lipid substrate, revealing that saturation of the lipid chain precedes biphytanyl formation. This work defines the remaining unannotated step in archaeal lipid biosynthesis and establishes the biosynthetic route for biphytanyl chain formation.

## GDGT–MAS binds a lipid substrate

**Fig. 2:**
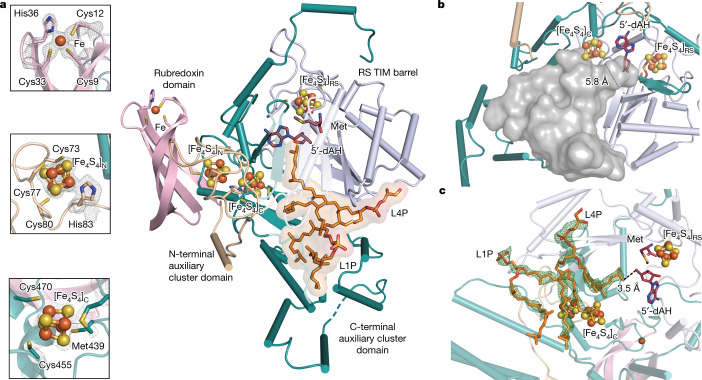
X-ray crystal structure of GDGT–MAS with bound cofactors and archaeal lipid substrate. **a**, Overall architecture of GDGT–MAS showing the rubredoxin domain (light pink), the N-terminal auxiliary cluster domain (wheat), the RS core domain (light blue) and the C-terminal auxiliary cluster domain (teal). The GDGT–MAS archaeal lipid complex contains one Fe ion, three [Fe_4_S_4_] clusters, methionine, 5′-dAH and two archaeal lipids: L1P and AG. Inlayed panels are 2*F*_*o*_–*F*_*c*_ maps contoured to 1.5σ, showing electron density for the three novel coordination spheres observed in the auxiliary metallocofactors. C_9_X_2_C_12_X_20_C_33_X_2_H_36_ coordination motif and the rubredoxin iron ion of the rubredoxin domain (top). [Fe_4_S_4_]_N_ cluster coordinated by the C_73_, C_77_, C_80_ and a conserved histidine (middle). Coordination sphere of the [Fe_4_S_4_]_C_ cluster with the labile Met439 in the Met-on configuration (bottom). TIM, triosephosphate isomerase. **b**, Cavity map of the GDGT–MAS active site reveals a hydrophobic pocket that narrows to a 5.8 Å-wide channel near the 5′-carbon of 5′-dAH. **c**, *F*_*o*_–*F*_*c*_ map contoured to 3.0σ, showing electron density for two archaeal lipids bound in the active site. The terminal carbon of AG is positioned 3.5 Å from the 5′-carbon of 5′-dAH, which suggests H• abstraction occurs on a terminal carbon of the phytanyl chain.

**Extended Data Fig. 2:**
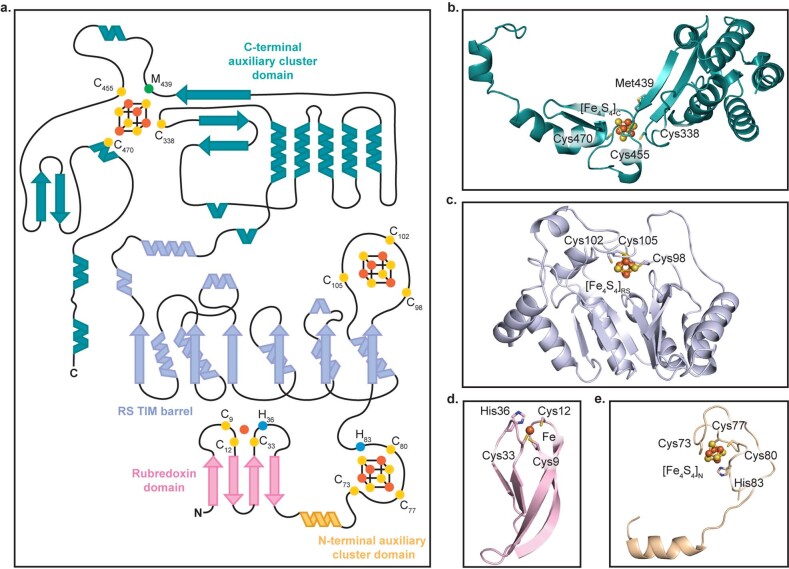
Topology and Ribbon diagrams of GDGT–MAS illustrating domain architecture. **a**, Topology diagram of GDGT–MAS. The dots represent atoms of cofactors and metal-coordinating residues. For the metallocofactors, the orange dots represent iron ions while the yellow dots indicate sulfide ions. On the peptide, cysteine ligands are modeled as yellow dots while the blue dots in the β turn of the rubredoxin domain and the loop region of the N-terminal auxiliary cluster domain represent histidine ligands. The green dot in the C-terminal auxiliary cluster domain is the labile methionine ligand, Met_439_. **b**–**e**, Ribbon diagram of **b**, C-terminal auxiliary cluster domain (teal), **c**, RS core or ¾ TIM barrel domain (light blue), **d**, rubredoxin domain (light pink), and **e**, N-terminal auxiliary cluster domain (wheat).

**Extended Data Fig. 3:**
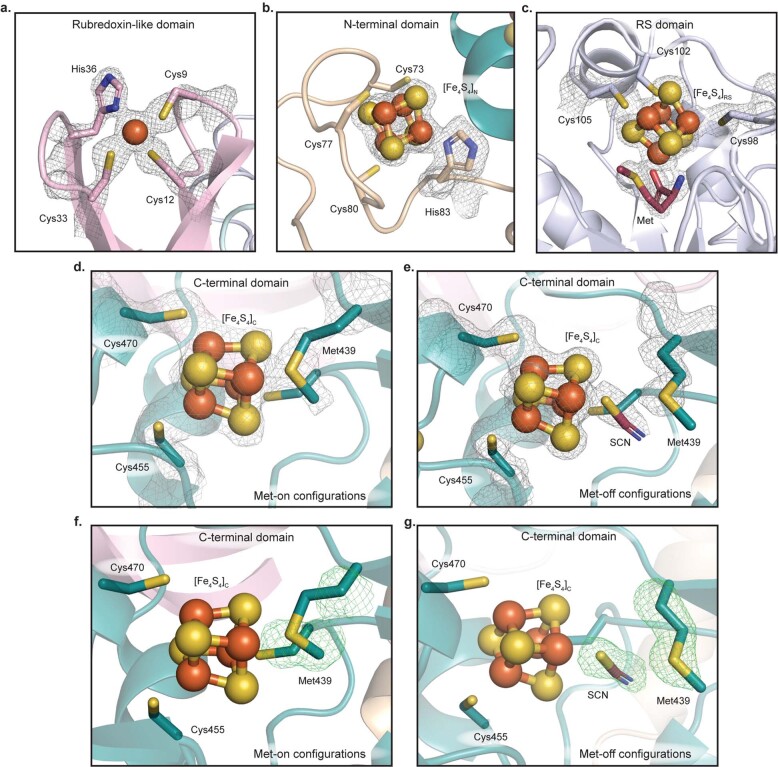
Coordination environment of the RS [Fe_4_S_4_] cluster and the three novel metallocofactors observed in GDGT–MAS. **a**, 2F_o_-F_c_ map contoured to 1.5σ (gray mesh) showing electron density for the rubredoxin coordination motif, C_9_X_2_C_12_X_20_C_33_X_2_H_36,_ and the rubredoxin iron ion of the rubredoxin domain. **b**, The novel coordination sphere observed in the N-terminal auxiliary [Fe_4_S_4_] ([Fe_4_S_4_]_N_) cluster ligated by C_73_, C_77_, C_80_ and a conserved histidine. Electron density is shown with 2*F*_*o*_*-F*_*c*_ map contoured to 1.5σ (gray mesh). **c**, 2*F*_*o*_*-F*_*c*_ map contoured to 1.5σ (gray mesh) showing electron density of the [Fe_4_S_4_]_RS_ cluster with Met bound to the unique iron. **d–e**, 2*F*_*o*_*-F*_*c*_ map contoured to 1.5σ showing electron density for the coordination sphere of the [Fe_4_S_4_]_C_ cluster with the labile Met439 ligand in the Met-on (**d**, observed in 7TOM) or Met-off (**e**, observed in 7TOL) configurations. In the Met-off state, the uncoordinated iron ion of the [Fe_4_S_4_]_C_ cluster is ligated by a reagent in the crystallographic condition, thiocyanate (SCN). **f–g**, *F*_*o*_*-F*_*c*_ omit map contoured to 3.0σ showing electron density for the labile Met439 ligand in the Met-on (**f**) or Met-off (**g**) configurations.

**Extended Data Fig. 4:**
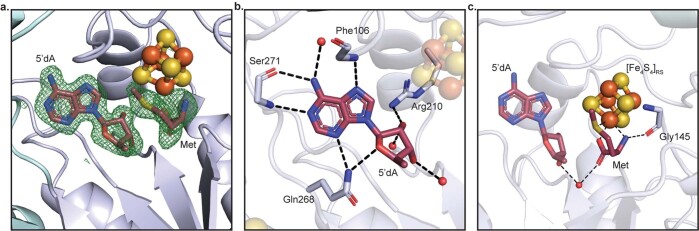
Binding of 5′-dAH and methionine to the RS [Fe_4_S_4_]. **a**, *F*_*o*_*-F*_*c*_ omit map contoured to 3.0σ (green mesh) showing electron density for 5′-dAH and methionine. **b**, Network of H-bonds that orchestrate binding of 5′-dAH. The adenine moiety of 5′-dAH is recognized by four H-bonds and the ribose moiety makes two H-bonds with four residues (Phe106, Arg210, Gln268, and Ser271) of the RS core domain. As observed in other structures of RS enzymes, N^7^ of adenine interacts with a nitrogen from the peptide backbone (Phe106) in the loop that resides between β_1_ and α_1_ of the ¾ TIM barrel. This loop also contains the CX_3_CX_2_C RS motif. N^3^ of the adenine ring and the ribose ring oxygen H-bond with Gln268 while N^1^ and N^6^ H-bond to the peptide backbone of Ser271. Interestingly, we predict that Gln268 and Ser271 reside on a loop region that regulates the active site opening for SAM, 5′-dAH, and Met (shown in Extended Data Fig. 8) Additionally, binding of 5′-dAH is further stabilized by H-bonding with three structurally conserved waters that interact with residues Tyr195, Asp199, Val236, Thr238, Thr273, and Arg275 (not shown). **c**, In canonical RS fashion, methionine coordinates to the unique iron in a bidentate manner via the carboxylate and amine functional groups. Gly145 forms an H-bond with the amine moiety to orient the ligand. Additionally, a water bridge is formed between the carboxylate group and the ribose ring of 5′-dAH.

**Extended Data Fig. 5:**
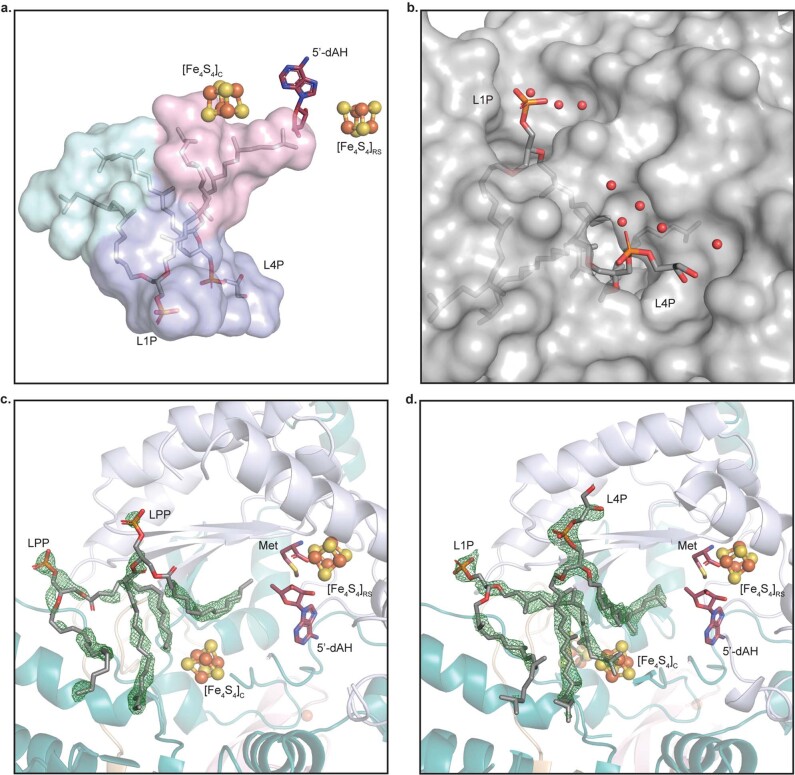
Lipid binding and electron density within the GDGT–MAS active site. **a**, The active site of GDGT–MAS contains two distinct hydrophobic pockets (colored light pink and cyan). GDGT–MAS directs one carbon chain from each lipid into separate hydrophobic pockets. The pocket that leads to 5′-dAH and the [Fe_4_S_4_]_C_ cluster (light pink) is the proposed reaction center of GDGT–MAS. **b**, GDGT–MAS binds the polar headgroup of lipids in a nonspecific fashion, which would allow the binding of lipids independent of the polar headgroups. The active site pockets that bind the headgroups do not make any direct H-bonds with L1P or L4P. Instead, an H-bonding network with several water molecules mediates binding of the headgroup to the protein. This large and versatile headgroup binding pocket would allow GDGT–MAS to accommodate larger polar headgroups, such as inositol, which suggests that GDGT–MAS catalyzes the formation GDGTs with varying headgroups. **c**, *F*_*o*_*-F*_*c*_ omit map of GDGT–MAS with bound bacterial lipids contoured to 3.0σ showing electron density that supports the modeling of two molecules of phosphatidic acid (1,2-dipalmitoyl-s*n*-glycero-3-phosphate, LPP). **d**, *F*_*o*_*-F*_*c*_ omit map of GDGT–MAS with bound archaeal lipids contoured to 3.0σ showing electron density of bound archaeol (2,3-di-*O*-phytanyl-s*n*-glycero-1-phosphate, L1P) and archaetidylglycerol (AG or L4P, 2,3-di-*O*-phytanyl-s*n*-glycero-1-phosphate-3′-s*n*-glycerol).

The discovery of GDGT–MAS arose from our efforts to characterize all subclasses of RS methylases^20,21,22^. GDGT–MAS was initially annotated as the pioneer enzyme—referred to as MJ0619—for the class D RS methylases subclass, and was proposed to methylate C7 and C9 on a pterin-like substrate during the biosynthesis of the methanopterin cofactor, which is an essential C1 carrier in methanogenic organisms^23^, ^24^ (Supplementary Fig. 1). However, we were unable to corroborate these findings. Therefore, we sought to determine the X-ray structure of GDGT–MAS to provide insight into the reaction that the enzyme catalyses. GDGT–MAS was isolated and crystallized under anoxic conditions in the presence of 5′-deoxyadenosine (5′-dAH) and methionine, and the structure was determined tο 1.85 Å resolution (Fig. 2a and Extended Data Table 1). GDGT–MAS contains three [Fe_4_S_4_] clusters and a mononuclear rubredoxin-type Fe^2+/3+^ cofactor, each found in a separate domain^25^ (Extended Data Figs. 2 and 3). A central RS domain contains a partial triosephosphate isomerase barrel fold and site-differentiated [Fe_4_S_4_] cluster that is common to all RS enzymes. Each iron of the RS cluster is coordinated by one of three cysteines in a CX_3_CX_2_C motif^26^. The remaining iron is open for coordination by the carboxylate and amino functional groups of SAM or methionine, the latter of which is bound here (Extended Data Fig. 4). The complex with 5′-dAH and methionine mimics the 5′-dA• intermediate state, allowing for delineation of the active site based on proximity to the 5′-carbon of 5′-dAH. In GDGT–MAS, this atom projects into a long hydrophobic substrate-binding pocket. This tunnel narrows to 5.8 Å and terminates at the 5′-carbon of 5′-dAH (Fig. 2b). The structure suggests that the initial annotation of GDGT–MAS as a pterin methylase is incorrect because binding of a bulky hydrophilic molecule, such as methanopterin, in the active site is unlikely. Instead, the structure suggests that H• abstraction would occur on a hydrophobic substrate, such as the long alkyl chain of a membrane lipid. In fact, we observed unmodelled electron density within the active site that could be reasonably interpreted as two molecules of phosphatidic acid (Extended Data Fig. 5c), presumably derived from the *Escherichia coli* overexpression system. The head groups of these bacterial lipids are oriented towards the exterior of the protein, with one alkyl chain from each lipid directed into two separate hydrophobic pockets (Extended Data Fig. 5). One pocket leads to 5′-dAH, suggesting that this pocket is the site where chemistry takes place. A second C-terminal auxiliary [Fe_4_S_4_] (designated [Fe_4_S_4_]_C_) cluster domain resides on the other side of this pocket and is near both lipid-binding sites (Extended Data Figs. 2, 3 and 5). The second lipid-binding pocket is composed of several amphipathic α-helices that position the hydrophobic residues towards the face of the pocket, a structural feature observed in other known lipid-synthesizing enzymes, such as GGR^26,27^ (Extended Data Fig. 5a and Supplementary Fig. 2). As a result, the second lipid pocket is lined solely by hydrophobic residues. Finally, N-terminal rubredoxin and N-terminal auxiliary [Fe_4_S_4_] (designated [Fe_4_S_4_]_N_) cluster domains are both located on the surface of GDGT–MAS (Extended Data Figs. 2 and 3). These features support the assignment of GDGT–MAS as a lipid-modifying enzyme.

**Extended Data Fig. 6:**
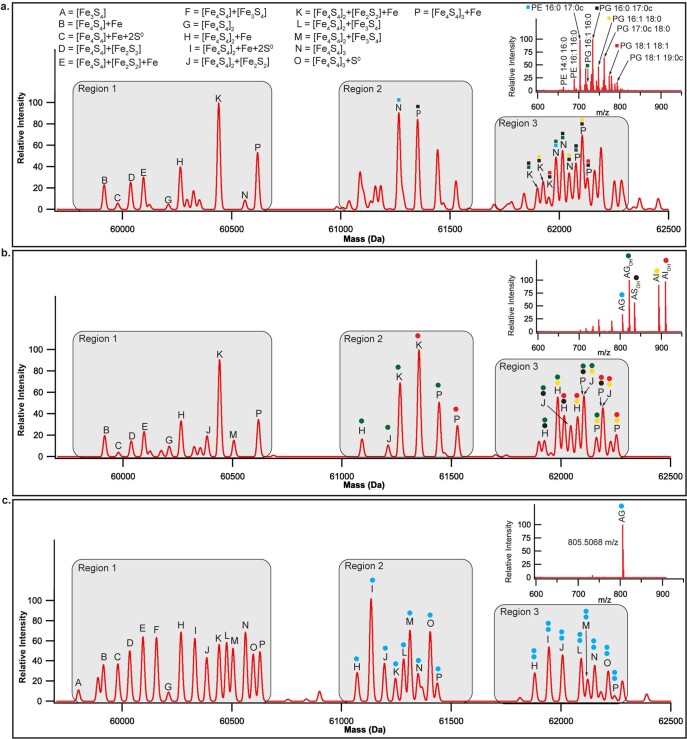
Native protein mass spectra of GDGT–MAS revealing apo, holo, and enzyme-ligand complexes. Panels **a**–**c** show the deconvoluted native protein mass spectra and high-resolution ejection mass spectra for **a**. as-isolated GDGT–MAS, **b**, *Ma* lipid-exchanged GDGT–MAS, **c**. AG lipid-exchanged GDGT–MAS. In each panel, the deconvoluted native protein mass spectra from 59,750 Da to 62,500 Da spans the bottom of the panel while the high-resolution ejection mass spectra of bound lipids are inlayed at the top right corner. Region 1 of the deconvoluted native protein mass spectra shows various iron-sulfur cluster states of GDGT–MAS from a single [Fe_3_S_4_] cluster (A) to the holoenzyme ([Fe_4_S_4_]_3_+Fe, P).* Region 2 displays GDGT–MAS–lipid complexes with a single lipid bound to the enzyme. Each peak represents a GDGT–MAS–lipid complex, wherein the letter identifies the observed iron-sulfur cluster state, and the colored dot indicates which lipid is bound. Similarly, Region 3 displays GDGT–MAS–lipid complexes with two bound lipids. Finally, the inlayed ejection mass spectra show the molecules bound to the enzyme-ligand complex. The observed *m/z* values from as-isolated GDGT–MAS, **a**, are consistent with the most prevalent phospholipids in *E. coli*. Comparably, the observed *m/z* values from *Ma* lipid-exchanged GDGT–MAS, **b**, are consistent with the most prevalent lipids in *Ma*. Finally, after lipid exchange with the AG substrate (blue dot), **c**, the full-scan MS of the native protein solely displays mass shifts associated with the binding of one or two AG molecules. Moreover, ejection of the bound lipids yielded a high-resolution mass spectrum containing an *m/z* value that matches AG. *The various iron-sulfur cluster states observed are presumed to be formed in the mass spectrometer during ionization. We have yet to identify an optimal ionization energy/technique to solely observe the holoenzyme.

Native protein mass spectrometry (full MS and tandem MS/MS) of GDGT–MAS was performed to determine whether phospholipids are bound in the active site of the enzyme^28^. The full-scan mass spectrum of as-isolated GDGT–MAS overexpressed in *E. coli* reveals a holoenzyme mass (60,618.38 AMU) consistent with the presence of one rubredoxin iron and three [Fe_4_S_4_] cluster metallocofactors (Extended Data Fig. 6a). Moreover, ejection of the lipids from the GDGT–MAS active site in the collision cell and subsequent analysis by high-resolution MS results in *m*/*z* values consistent with the most prevalent phospholipids in *E. coli*. The content of the bound phospholipid was then estimated using *E. coli* cyclopropane fatty acid synthase (CFAS), a lipid-modifying enzyme that is isolated with a bound phospholipid^29^. The lipid levels found in GDGT–MAS were similar to those found in CFAS, suggesting that the electron density of the active site in the GDGT–MAS structure arises from two bacterial phospholipids.

The finding of well-ordered *E. coli* phospholipids in the GDGT–MAS active site suggested that the true substrate for the enzyme is archaeal lipids. To verify that the enzyme binds archaeal lipids, the protein was incubated at 40 °C with a lipid extract from a *Methanosarcina acetivorans* cell lysate to allow the exchange of archaeal lipids into the active site. Unbound lipids were removed by gel-filtration chromatography, and the resulting protein was characterized by native MS. Similar to that of as-isolated GDGT–MAS, the full mass spectrum of archaeal lipid-exchanged GDGT–MAS displays a holoenzyme mass consistent with the four metallocofactors (Extended Data Fig. 6b). The spectrum also displays mass shifts consistent with the predominate archaeal lipids observed in the lipid extract from *M. acetivorans* cell lysate (Δ821.6 and Δ909.7 from 3-hydroxyarchaetidylglycerol and 3-hydroxyarchaetidylinositol, respectively). In addition, ejection of the bound lipids yielded a high-resolution mass spectrum exhibiting *m*/*z* values of 821.6 and 909.7. These mass shifts indicate that GDGT–MAS binds two archaeal lipids.

To obtain a more precise picture of the GDGT–MAS active site and a better understanding of the reaction that the enzyme catalyses, we determined a 2.05 Å resolution structure of the *M. acetivorans* lipid-exchanged GDGT–MAS in the presence of 5′-dAH + methionine. As expected, the active site contained electron density that was confidently modelled as two archaeal lipids—archaeol (2,3-di-*O*-phytanyl-*sn*-glycero-1-phosphate (L1P)) and archaetidylglycerol (AG (also known as L4P, 2,3-di-*O*-phytanyl-*sn*-glycero-1-phosphate-3′-*sn*-glycerol))—in the lipid-binding pockets identified previously (Fig. 2c). The archaeal lipid chain extends through the hydrophobic channel into the active site, positioning the terminal carbon 3.5 Å from the 5′-carbon of 5′-dAH, a suitable distance for direct H• abstraction by a 5′-dA• (ref. ^26^). These observations suggested that GDGT–MAS might be the elusive enzyme that catalyses the formation of the biphytanyl chain during GDGT synthesis.

## Analysis of biphytanyl chain formation

**Extended Data Fig. 7:**
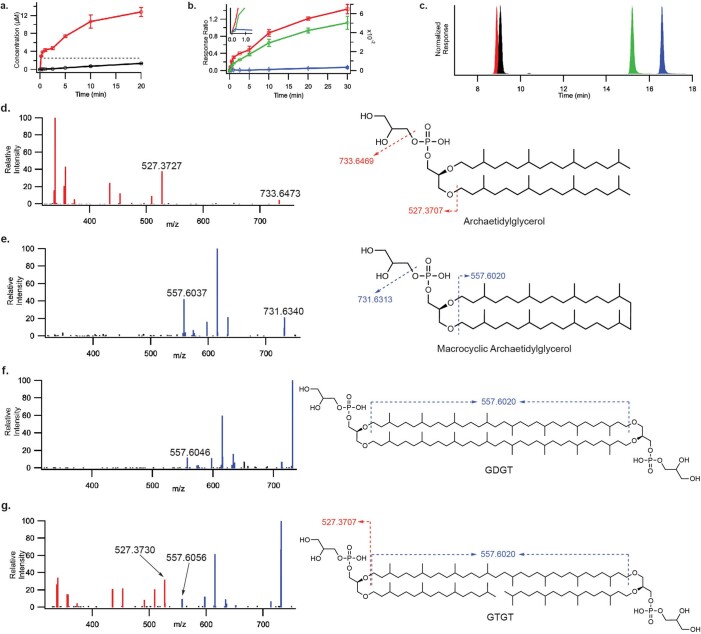
Identification and characterization of lipid products formed by GDGT–MAS. **a**–**c**, Time-dependent production of 5′-dAH and lipid products under catalytic conditions. Unless otherwise stated, GDGT–MAS in vitro activity assays were conducted with 2.5 µM GDGT–MAS, 10 µM archaetidylglycerol (AG), 300 µM SAM, 1 mM TiCitrate, 200 mM KCl, and 10 µM D-methionine-methyl-d_3_ in 75 mM HEPES, pH 7.5. **a**, Time-dependent formation of 5′-dAH in the presence (red line) and absence (black trace) of AG substrate. The dotted gray line indicates concentration of enzyme in the assay. **b**, Time-dependent formation of the three unknown lipids denoted lipid I (red trace), lipid II (green trace), and lipid III (blue trace). **c**, LC-MS extracted-ion chromatogram of the GDGT–MAS reaction showing retention time of AG substrate (black trace) at 9.1 min, lipid I at 8.9 min (red trace), lipid II at 15.2 min (green trace), and lipid III (blue trace) at 16.6 min. **d**–**g**, Structural characterization of AG (**d**), macrocyclic AG (**e**), GDGT (**f**), and GTGT (**g**) by tandem MS/MS with dashed lines representing the fragmented bond. Fragmentation of the ether bond on the AG substrate, shown in panel **d**, results in a 527.3727 m/z daughter ion, indicating the neutral loss of one phytanyl chain. Novel lipid I and lipid II, shown in panels **e** and **f**, respectively, produce similar fragmentation patterns, revealing the presence of a biphytanyl chain (557.6046 m/z daughter ion) that results from cleavage of two ether bonds. This result indicates that both lipid I and lipid II solely contain biphytanyl. **g**, Tandem MS/MS on novel lipid III produces a fragmentation pattern that indicates the molecule contains both a phytanyl chain and a biphytanyl chain, as evidenced by the m/z 527.3730 and 557.6056 daughter ions, respectively. Therefore, lipid III is GTGT. Error bars represent one standard deviation for reactions conducted in triplicate, with the center representing the mean. *See Supplementary Fig. 6 for complete fragmentation.

In vitro activity assays were performed to assess whether GDGT–MAS catalyses the formation of the biphytanyl chain. However, the ambiguity in the biosynthetic pathway suggests two potential substrates for the reaction: lipids containing geranylgeranyl (unsaturated) or phytanyl (saturated) chains. Our results from previous native-spray protein MS suggested that GDGT–MAS primarily binds saturated archaeal lipids, with 3-hydroxyarchaetidylglycerol being the predominant species. Moreover, in activity assays using *M. acetivorans* cell extract, the fully saturated lipids decline substantially in abundance as a function of time, whereas the unsaturated lipids do not (Supplementary Fig. 3). These results suggest that the substrate for GDGT–MAS contains fully saturated phytanyl chains. However, in contrast to archaeol lipids from *M. acetivorans*, hydroxylation at C3 of the phytanyl chain has not been observed in *M. jannaschii*, the organism that produces GDGT–MAS^30,31^. Therefore, we synthesized saturated AG—a lipid found in *M. jannaschii*—to be used as the substrate and monitored the reaction by liquid chromatography–MS^32^ (Extended Data Fig. 7a–c). In Extended Data Fig. 7a, the time-dependent formation of 5′-dAH is shown, which reflects the reductive cleavage of SAM, an indicator of radical chemistry. The rate of 5′-dAH formation in the presence of the AG substrate (red trace) is considerably enhanced over that in its absence (black trace), which reflects abortive cleavage of SAM. As shown in Extended Data Fig. 7a, a burst of 5′-dAH is observed, which is followed by a slower phase of 5′-dAH formation during the following 20 min. This burst of 5′-dAH triggered by the presence of AG suggests that chemistry is taking place on the lipid substrate.

Lipid products were also profiled throughout the GDGT–MAS assay by high-resolution MS (electrospray ionization in negative mode) to elucidate the reaction performed by GDGT–MAS. The high-resolution mass spectrum for the AG substrate exhibits *m*/*z* of 805.6696. In reactions lacking SAM or GDGT–MAS, the intensity of this peak remained the same and no new peaks were observed. By contrast, under full turnover conditions, three new peaks appeared in a time-dependent manner. The first peak (lipid I; Extended Data Fig. 7c, red trace), eluting at 8.9 min, exhibited *m*/*z* of 803.6509, a shift in mass corresponding to the loss of 2 H• from the AG substrate. The second peak (lipid II; Extended Data Fig. 7c, green trace), eluting at 15.2 min, exhibited *m*/*z* of 1,608.3141, which is consistent with the chemical formula C_92_H_185_O_16_P_2_^−^, indicating dimerization of the AG substrate with the corresponding loss of 4 H•. Finally, the third peak (lipid III; Extended Data Fig. 7c, blue trace), eluting at 16.6 min, exhibited *m*/*z* of 1,610.3293, consistent with the chemical formula C_92_H_187_O_16_P_2_^−^, indicating dimerization of the AG substrate with the corresponding loss of 2 H•.

These observations suggest that lipid III results from forming one C–C bond between two sp^3^-hybridized carbons from two AG molecules. Correspondingly, lipid II results from forming two C–C bonds between two AG molecules. Finally, lipid I results from forming one intramolecular C–C bond between two phytanyl chains of one AG molecule. In addition, monitoring lipid production as a function of time reveals that the formation of lipid III (blue trace) proceeds with a small burst that is followed by an immediate decay, whereas lipid II (green trace) continues to accumulate (Extended Data Fig. 7b). This behaviour suggests that lipid III is an intermediate in the formation of lipid II, indicating the sequential formation of two biphytanyl chains. Therefore, the formation of the first biphytanyl chain yields glycerol trialkyl glycerol tetraether (GTGT; lipid III), showing *m*/*z* of 1,610.3293, whereas the formation of the second biphytanyl chain yields the final tetraether product, GDGT (lipid II), showing *m*/*z* of 1,608.3141. Lipid I (red trace) exhibits the chemical formula C_46_H_92_O_8_P^−^ (*m*/*z* of 803.6509), which is 2 H• less in mass than that of the AG substrate. Lipid I accumulates throughout the reaction, indicating that it is an additional product, which we suggest is the macrocyclic archaeol.

Tandem MS/MS was used to confirm the identities of the three products of the GDGT–MAS reaction^33^. MS/MS was performed in positive-ion mode to obtain definitive fragmentation patterns that would allow unambiguous determination of biphytanyl chain formation. Therefore, all lipids contain an additional two protons and exhibit a mass of 2.0146 AMU greater than they would in negative-ion mode. At a normalized collision cell energy of 20 eV, we observed a diagnostic fragmentation occurring across the ether bond, resulting in the neutral loss of one phytanyl chain and a daughter ion of 527.3707 *m*/*z* (Extended Data Fig. 7d). Tandem MS/MS of lipid I and lipid II results in a distinct fragmentation pattern (between *m*/*z* values of 300 and 700) from that observed for AG, with a daughter ion of 557.6020 *m*/*z* (Extended Data Fig. 7e,f). This daughter ion has the chemical formula C_40_H_77_^+^ and can only result from the fragmentation of a parent molecule containing a biphytanyl chain, showing definitively that GDGT–MAS catalyses the formation of C–C bonds during biphytanyl chain biosynthesis^33^ (Extended Data Fig. 7g). Moreover, tandem MS/MS of lipid II results in a fragmentation pattern containing both daughter ions (*m*/*z* of 527.3707 and 557.6020), indicating the presence of both phytanyl and biphytanyl chains. Therefore, tandem MS/MS of the unknown lipids reveals that GDGT–MAS catalyses the formation of the biphytanyl chain during the biosynthesis of the macrocyclic archaeol (lipid I) and GDGT (lipid II), in which GTGT (lipid III) is an intermediate in the biosynthesis of GDGT.

## Insight into the GDGT–MAS reaction

**Fig. 3:**
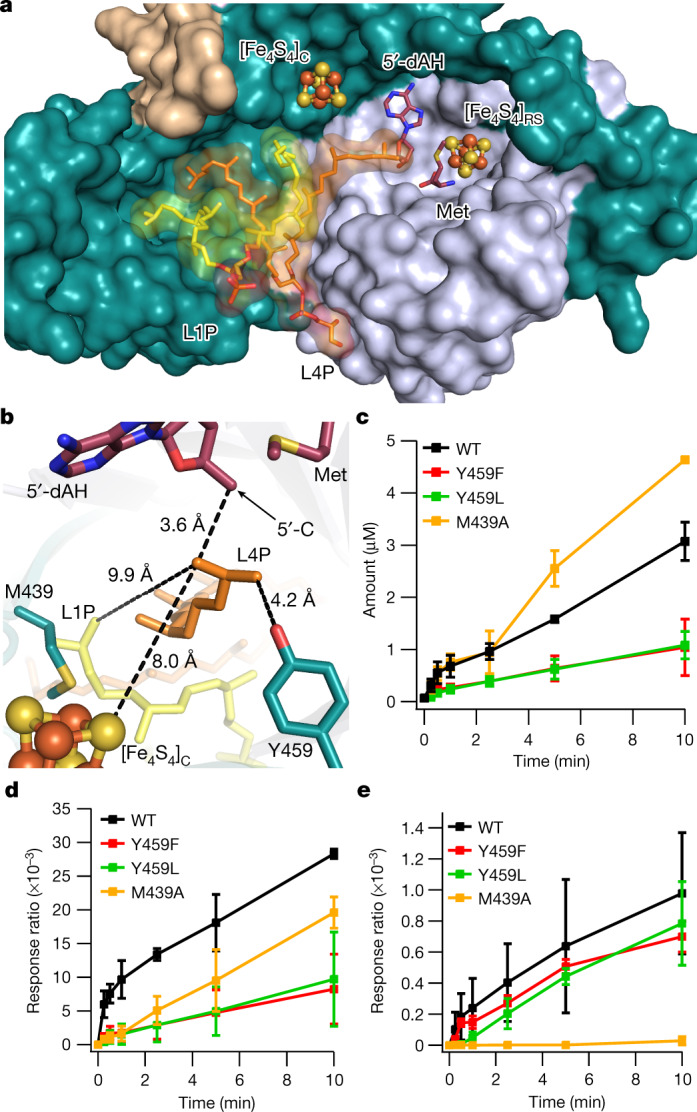
Structural insight into the mechanism of biphytanyl chain formation. **a**, The active site of GDGT–MAS binds two archaeal lipids, L1P (yellow) and AG (orange), and directs one carbon chain from each lipid into separate pockets. The pocket that leads to 5′-dAH and the [Fe_4_S_4_]_C_ cluster is the proposed reaction centre of GDGT–MAS. **b**, GDGT–MAS catalyses the formation of the biphytanyl chain by coupling two terminal Csp^3^ carbons, which our substrate-bound structure indicates are 9.9 Å apart. Generation of the Csp^3^–Csp^3^ bond would necessitate two sequential C–H activations and storage of a high-energy substrate radical intermediate. The substrate-bound complex suggests two potential mechanisms for storage of the high-energy radical intermediate: formation of (1) a terminal olefin and (2) an [Fe_4_S_4_]_C_-substrate intermediate. In the first mechanism, substrate radical formation leads to loss of an electron and a proton to yield a terminal olefin intermediate on one chain. Tyr459 is 4.2 Å away from the terminal carbon of the substrate and is strictly conserved, suggesting that tyrosinate might facilitate this proposed mechanism. In the second mechanism, the substrate radical might couple with the [Fe_4_S_4_]_C_ cluster to yield an S–C bond intermediate. The sulfur atom of the [Fe_4_S_4_]_C_ is 8.0 Å from the high-energy substrate radical intermediate. **c**–**e**, Time-dependent production of 5′-dAH (**c**), mAG (**d**) and GDGT (**e**) of in vitro activity assays containing either wild-type or mutant (Y459F, Y459L and M439A) forms of GDGT–MAS. These mutagenesis experiments reveal that Y459 does not have an essential role in the GDGT–MAS mechanism, which suggests that the radical intermediate is stabilized by interaction with the [Fe_4_S_4_]_C_ cluster. The error bars represent one standard deviation for reactions conducted in triplicate, with the centre representing the mean.

**Extended Data Fig. 8:**
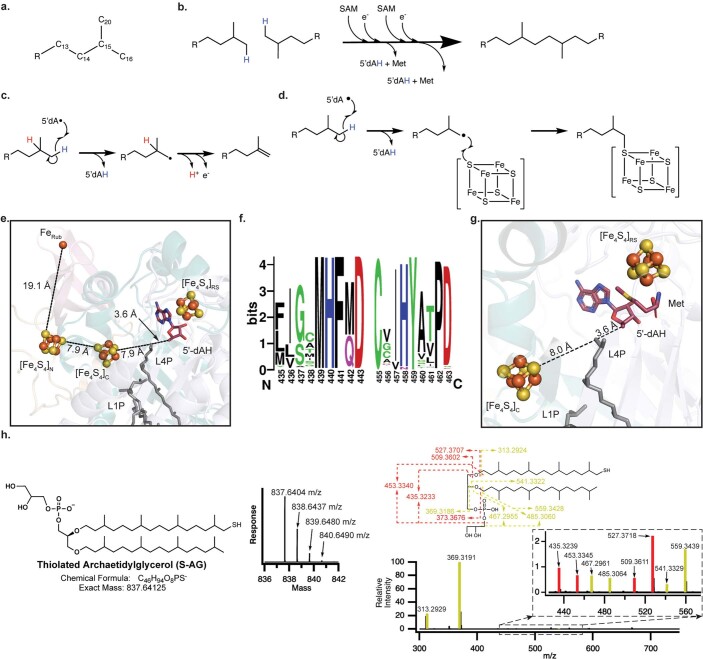
Structural and biochemical evidence for an AG–[Fe_4_S_4_]_C_ intermediate. **a**, Carbon numbering of the phytanyl chain. C16 and C20 are the phytanyl terminal carbons. **b**, Proposed stoichiometry for the reaction catalyzed by GDGT–MAS, wherein the formation of the biphytanyl chain requires two molecules of SAM. The catalyzed reaction requires storage of a high-energy radical intermediate, which can be achieved by (**c**) olefin formation or (**d**) creation of a sulfur-carbon bond. **e**, GDGT–MAS contains three auxiliary metallocofactors that might play a role in tuning redox potentials and electron transfer. **f**, Sequence alignment of 1000 archaeal GDGT–MAS homologs indicates that the labile Met439 and Tyr459 are completely conserved. Sequence alignment is visualized in Weblogo format. **g**, The terminal carbon of the phytanyl chain is 8.0 Å from the [Fe_4_S_4_]_C_, which is a favorable distance for coupling of the substrate radical with the cluster to generate a sulfur-carbon bond intermediate. **h**, Structural characterization of the sulfur-carbon bond intermediate (sulfur-containing archaetidylglycerol, S-AG) with the structure, chemical formula, and exact mass on the left of the panel. The mass spectrum in the center shows the m/z values observed for S-AG that result from natural abundance isotopes. The product ions resulting from tandem MS/MS fragmentation of S-AG are shown at the right of the panel with dashed lines identifying the location of the fragmented bond and the resulting theoretical m/z. All observed m/z values from tandem MS/MS are displayed on the mass spectrum. In the tandem MS/MS spectra, dashed lines and product ions colored red indicate the presence of a phytanyl chain, while yellow coloring indicates the presence of a sulfur-containing phytanyl chain.

GDGT–MAS forms the biphytanyl chain from substrates containing fully saturated lipids, indicating the formation of a C–C bond between two inert sp^3^-hybridized carbon centres. This reaction necessitates two H• abstractions to generate two substrate radicals, which we postulate are mediated by two sequentially generated 5′-dA•. Therefore, two molecules of SAM are needed to construct one Csp^3^–Csp^3^ bond. However, how the enzyme stabilizes the first substrate radical while generating the second substrate radical to allow for Csp^3^–Csp^3^ bond formation was as yet unknown. Two potential strategies can be envisioned (Fig. 3a,b). In the first strategy, substrate radical formation leads to the loss of an electron and a proton (perhaps facilitated by Tyr459) to yield a terminal olefin intermediate on one chain. In the next step of this mechanism, a second substrate radical attacks the terminal olefin, resulting in the formation of the C–C bond (Fig. 3b and Extended Data Fig. 8). Tyr459 is 4.2 Å away from the terminal carbon of the substrate and is strictly conserved across 1,000 archaeal GDGT–MAS homologues, suggesting that it might have an essential role in catalysis. In the second strategy, the substrate radical might couple with the [Fe_4_S_4_]_C_ cluster, which potentially contains an iron ion with an available coordination site. In the next step, the second substrate radical would attack the carbon atom bound to the cluster, resulting in the formation of a C–C bond. The [Fe_4_S_4_]_C_ is 8.0 Å from C16 of the phytanyl chain, which is close enough for the transfer of an electron from the substrate radical intermediate to [Fe_4_S_4_]_C_, or perhaps even close enough for [Fe_4_S_4_]_C_–substrate bond formation (Fig. 3b). To distinguish between the two aforementioned strategies, activity assays were first performed with Y459F and Y459L variants. If the formation of an olefin intermediate is the operative mechanism, then these substitutions should disrupt H• abstraction from the substrate and therefore abolish turnover, given that Tyr459 is the only ionizable amino acid residue suitably positioned to perform the role of a general base. As shown in Fig. 3c–e, these variants exhibit robust activity, producing both macrocyclic archaetidylglycerol (mAG) and GDGT. These results suggest that a terminal olefin—at least through deprotonation by Tyr459—is not an intermediate in the GDGT–MAS reaction.

## Identification of a novel lipid species

**Fig. 4:**
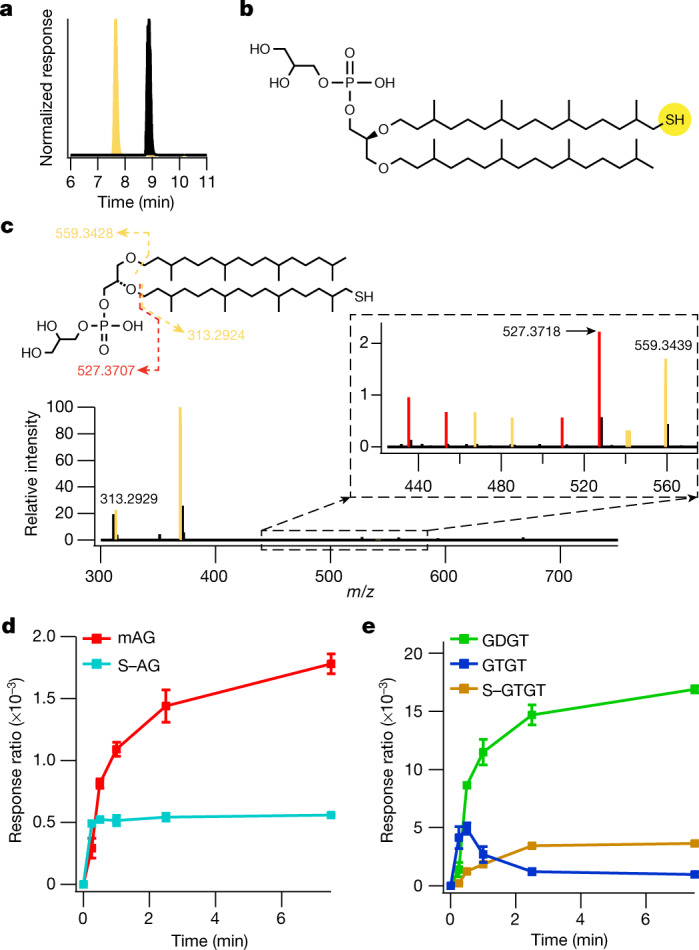
Stabilization of a high-energy radical intermediate via S–C bond formation. **a**, Liquid chromatography–MS extracted-ion chromatogram of the GDGT–MAS reaction showing retention time of AG substrate (black trace) at 9.1 min and lipid IV (yellow trace) at 7.8 min. **b**, Structure of thiolated AG (lipid IV, S–AG). **c**, Structural characterization of S–AG by tandem MS/MS, with dashed lines representing the fragmented bond. Fragmentation of S–AG predominantly cleaves the ether bond, resulting in a 313.2929 *m*/*z* daughter ion, indicating the observation of a thiolated phytanyl chain. In addition, less favoured fragmentation patterns (present in the dashed box) reveal a similar fragmentation pattern to the AG substrate, in which the presence of the 527.3718 *m*/*z* daughter ion indicates the neutral loss of the thiolated phytanyl chain, whereas the 559.3439 *m*/*z* daughter ion indicates the neutral loss of the phytanyl chain. Yellow fragments indicate sulfur-containing daughter ions of S-AG; red fragments are nonsulfur-containing daughter ions of S-AG. **d**,**e**, Time-dependent production of the S–C bond intermediates, S–AG and S–GTGT, observed during in vitro activity assays with wild-type GDGT–MAS and limiting SAM, suggests that S–AG is the intermediate for mAG and GTGT synthesis and that S–GTGT is the intermediate for GDGT synthesis. Production of S–AG (teal trace) compared with the formation of mAG (red trace) (**d**), and production of S–GTGT (brown trace) from GTGT (blue trace) towards the formation of GDGT (green trace) (**e**) are shown. The error bars represent one standard deviation for reactions conducted in triplicate, with the centre representing the mean.

To ensure that an olefin intermediate is not formed through another mechanism, perhaps involving an unidentified general base, activity assays were also performed under limiting SAM concentrations. Both aforementioned mechanisms predict that two molecules of SAM are required to form one C–C bond. One molecule of SAM is required to generate the potential olefin intermediate, whereas the second molecule of SAM is necessary to complete the reaction, generating the C–C bond (Extended Data Fig. 8). Therefore, activity assays performed in the presence of 1 equiv of SAM with respect to enzyme might be expected to favour the accumulation of the olefin intermediate to detectable levels. Reactions were performed using 24 µM SAM and 30 µM GDGT–MAS that had been pre-loaded with the synthetic AG lipid using the aforementioned lipid-exchange procedure. As expected, on the basis of our observations with the Tyr459 variants, no olefin-containing species was detected. Unexpectedly, however, we observed the accumulation of a new lipid (lipid IV; Fig. 4a, yellow trace), eluting at 7.8 min, that exhibits an *m*/*z* of 837.6404, a shift in mass corresponding to the addition of one sulfur atom to the AG substrate. Tandem MS/MS was used to elucidate the structure of the sulfur-containing AG (lipid IV, S–AG; Fig. 4b,c). Similar to the fragmentation pattern observed for the AG substrate, we observed a daughter ion of 527.3707 *m*/*z*, which indicates fragmentation of the ether bond resulting in the neutral loss of the sulfur-containing phytanyl chain. We observed a diagnostic fragmentation occurring across the same ether bond, resulting in a daughter ion of 313.2929 *m*/*z*. This second daughter ion has the chemical formula C_20_H_41_S^+^ and can only result from the fragmentation of a parent molecule with a sulfur-containing phytanyl chain, showing definitively that a S–C bond is formed on a phytanyl chain during the GDGT–MAS reaction.

**Fig. 5:**
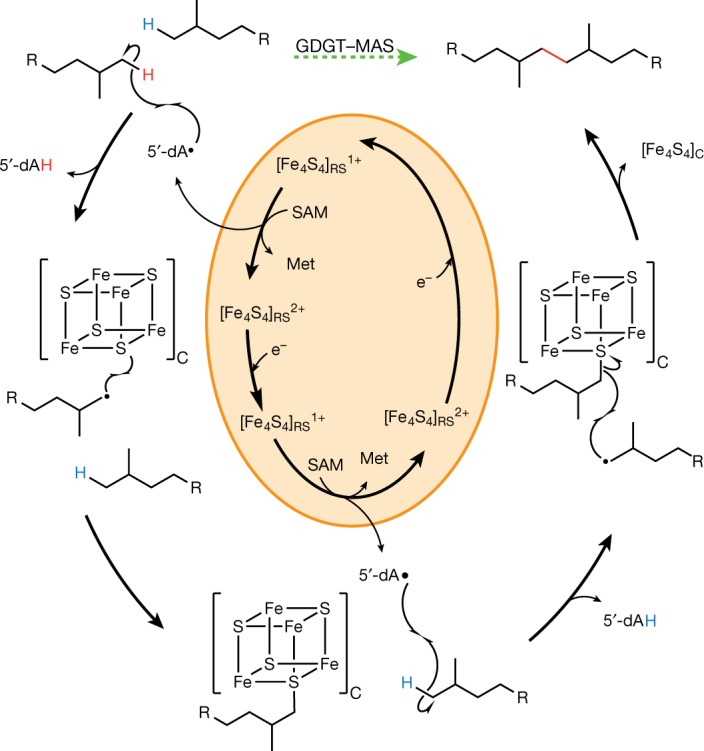
Proposed reaction mechanism for the formation of the biphytanyl chain. Formation of the biphytanyl chain from two phytanyl chains (R = remaining molecule of archaeol; Fig. 1b.) The orange circle indicates the chemistry performed by the [Fe_4_S_4_]_RS_. The dashed green arrow indicates the reaction catalysed by GDGT–MAS, with the C–C bond formed during the formation of the biphytanyl chain highlighted in red.

**Extended Data Fig. 9:**
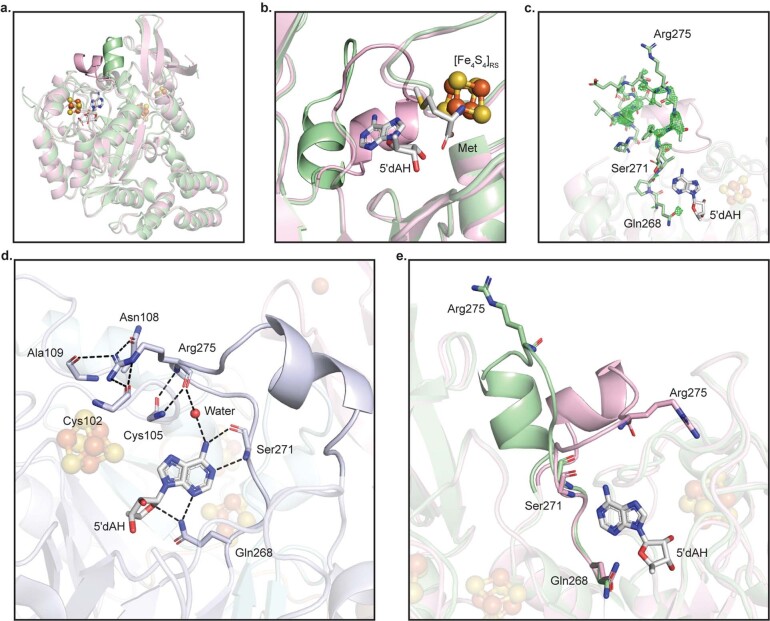
The ¾ TIM barrel loop domain hypothesized to mediate active site opening for SAM, 5′-dAH, and methionine. Overlay of structures determined by X-ray crystallography (light pink) with AlphaFold predicted model (pale green) highlighting a loop region (Gln268 through Arg285, not transparent) that opens the top of the *RS* ¾ TIM barrel to solvent. **a**, view from exterior of protein. **b**, view from interior of protein. **c**, Analysis of F_o_-F_c_ omit map contoured to 3.0σ (green mesh) in the GDGT–MAS archaeal lipid complex shows electron density for the AlphaFold predicted loop. Therefore, the X-ray crystal structure has partial occupancy for the open loop. However, the major confirmation within the protein crystal lattice is closed. **d**, These structures suggest that when Gln268 and Ser271 form H-bonds with the adenosine and ribose moieties of 5′-dAH—and presumably SAM—the loop closes and traps a key molecule of water. This water molecule links N^6^ of 5′-dAH and the peptide backbone of Arg275 through H-bonding, which positions Arg275 into a H-bonding network with Cys102, Cys105, Asn108, and Ala109 to close the active site from solvent. **e**, Conformational difference between Gln268, Ser271, and Arg275 in the open (pale green) versus closed (light pink) loop. ^*^Refer to Supplementary Fig. 6 for C-alpha structural alignments.

The identification and structural characterization of S–AG suggests that the high-energy substrate radical intermediate formed through H• abstraction by the 5′-dA• is stabilized by coupling with [Fe_4_S_4_]_C_ to yield an intermediate S–C bond (Fig. 5). The detection of the sulfur-containing lipid results from the inability of a large fraction of enzyme to complete the reaction because of the absence of the second required molecule of SAM. The sulfur-containing product is then liberated upon acid treatment of the enzyme, which is known to degrade iron–sulfur (FeS) clusters. Our assays under limiting SAM concentrations also revealed the presence of mAG, GTGT, GDGT and the molecule S–GTGT, which exhibits a chemical formula of C_92_H_187_O_16_P_2_S^−^ and *m*/*z* of 1,642.3020 (Fig. 4d,e). These results suggest that, although GDGT–MAS is in slight excess over SAM, some enzyme molecules nonetheless use multiple equiv of SAM before others are able to react. The mAG and GTGT products would necessitate 2 equiv; the S–GTGT product would necessitate 3 equiv; and the GDGT product would necessitate 4 equiv. Our finding of the S–GTGT product is consistent with it being an intermediate en route to GDGT. How the products of the first SAM cleavage exit the active site to allow the second SAM to bind is not known. However, the structure of GDGT–MAS suggests a conformational change that would permit the dissociation of 5′-dAH and methionine and binding of a second equivalent of SAM without dissociation of the lipid substrates (Fig. 3a and Extended Data Fig. 9).

Finally, the [Fe_4_S_4_]_C_ contains a labile methionine ligand (Met439), which our structures have captured in both the ligated (Met-on) and the unligated (Met-off) states (Extended Data Fig. 3). A M439A variant was constructed and used in activity assays to assess the importance of this amino acid residue in catalysis. The variant still exhibited robust activity (Fig. 4c–e), suggesting that the methionine ligand may be present simply to maintain cluster integrity in the absence of substrate. Several enzymes that form S–C bonds to sulfur atoms within FeS clusters have non-traditional ligands to the cluster, such as an Arg residue in biotin synthase^34^, a Ser residue in lipoyl synthase^35,36^ and a pentasulfide bridge in RimO and MiaB^37,38^. Although the Ser residue is important in the lipoyl synthase reaction, the Arg residue in biotin synthase can be substituted with various amino acid residues with no notable loss of activity^39^. The importance of the pentasulfide bridge in RimO and MiaB has not yet been established. At present, it is not clear what roles the remaining metallocofactors have in the reaction.

## Discussion

In this work, we identified—as have others recently^14^—the elusive enzyme responsible for generating the macrocyclic ether-linked lipids found predominantly in Archaea but also in several species of bacteria. Moreover, we determined the structure of the enzyme in the presence of all of its metallocofactors and in the absence and presence of its lipid substrate, and provided evidence for an unprecedented use of an FeS cluster for Csp^3^–Csp^3^ bond formation. *M. jannaschii* contains both mAG and GDGT, and our studies showed that GDGT–MAS synthesizes both macrocyclic lipids seemingly from the same active site (Supplementary Fig. 4). Currently, we do not understand the factors that govern the partitioning between the two lipid products, some of which might be temperature related. Our in vitro activity assays clearly show that C–C bond formation occurs between two fully saturated lipid chains, ruling out an alternative unsaturated pathway and resolving a long-standing conundrum in the field. Although the enzyme might catalyse macrocyclic lipid formation on substrates containing unsaturated bonds due to inefficient reduction of phytanyl chains by GGR, there is no mechanistic advantage for doing so given that unsaturated carbons are not intermediates in the reaction. Our findings, and those of Zeng et al.^14^, now provide a strong basis for the use of GDGT as a biomarker in paleoenvironmental indices. Identification of the genetic link for biphytanyl chain formation enables future work to understand which organisms contribute to the GDGT pools. In addition, a sequence similarity network generated from the GDGT–MAS InterPro protein family (IPR034474) indicates that GDGT–MAS homologues exist in bacterial organisms that are not known to synthesize biphytanyl chains (Supplementary Fig. 5). Our studies provide a roadmap for investigating the reactions catalysed by these enzymes and their physiological roles. Furthermore, our findings expand the scope of reactivity within the RS superfamily to include Csp^3^–Csp^3^ cross-coupling, a reaction that, to our knowledge, has not been observed in nature outside of C–C bond formation by methylation of inert carbon centres^20,40–43^.

## Methods

### Plasmid construction of pMj0619

The gene encoding *M. jannaschii* GDGT–MAS (*mj0619*; UniProt ID: HMPTM_METJA) was optimized for expression in *E. coli* and ordered from Invitrogen GeneArt Gene synthesis with an added 5′ NdeI cut site and a 3′ XhoI cut site. The gene encoding GDGT–MAS was removed from the GeneArt pMA-T vector by digestion with the NdeI and XhoI restriction enzymes and subsequently ligated into linearized pET28a plasmidusing T4 DNA ligase. The resulting plasmid was named pMj0619. *E. coli* DH5α cells were transformed with pMj0619, and the sequence was confirmed by DNA sequencing at Pennsylvania State Genomics Core Facility.

### Overexpression and purification of GDGT–MAS

Expression and purification of GDGT–MAS was modified from previously established methods used to obtain soluble RS enzymes via heterologous expression in *E. coli*^44,45^. An *E. coli* BL-21(DE3) strain with the pDB1282 and pBAD42-BtuCEDFB plasmids was transformed with pMj0619. A single colony of the resulting construct was used to inoculate 200 ml LB medium starter culture containing 50 μg ml^−1^ kanamycin, 50 μg ml^−1^ spectinomycin and 100 μg ml^−1^ ampicillin. The starter culture was incubated overnight at 37 °C and shaken at 250 rpm. A 4 ml aliquot of the starter culture was used to inoculate 4 l of ethanolamine minimal medium, containing equivalent antibiotic concentrations, in a non-baffled 6-l Erlenmeyer flask and grown at 37 °C (ref. ^45^). At an OD_600_ = 0.6, arabinose was added to the culture to a final concentration of 0.2% (w/v) to induce expression of the *isc* and *btu* operons on pDB1282 and pBAD42-BtuCEDFB, respectively. Simultaneously, 25 µM FeCl_3_ was added to the medium as the iron source for FeS cluster biogenesis. The cultures were then grown to an OD_600_ = 1.0, and 50 μM IPTG was added to the growth to induce the expression of GDGT–MAS. The temperature was reduced to 30 °C and the culture was incubated for 5 h before harvesting the cells by centrifugation at 7,000*g*. The harvested cells were flash-frozen and stored at −80 °C until protein purification. For reasons that we do not understand, the use of the pBAD42-BtuCEDFB plasmid greatly increased the solubility and yield of MJ0619. Although the plasmid was generated to enhance the solubility of cobalamin-containing proteins, it has also been found to enhance the solubility of some proteins that do not bind to cobalamin^46^.

All remaining steps were performed in a Coy Laboratories anaerobic chamber or in an airtight vessel to ensure an oxygen-free environment. Cell paste was resuspended in lysis buffer (50 mM HEPES, pH 7.5, 300 mM KCl, 10% glycerol, 10 mM β-mercaptoethanol (BME) and 4 mM imidazole) for 10 min. The following reagents and enzymes were added to the resulting solution and allowed to incubate for 5 min: 1 mg ml^−1^ lysozyme, 0.1 mg ml^−1^ DNAse, 0.17 mg ml^−1^ PMSF, 0.8 μg ml^−1^ cysteine, 0.7 μg ml^−1^ FeCl_3_ and 0.2% Triton X-100. The cell suspension was then sonicated for a total of 5 min (45 s on, 7 min off) at 35% amplitude. The lysate was centrifuged at 45,000*g* and the resulting supernatant was loaded onto a Ni-NTA column equilibrated with approximately 100 ml lysis buffer. The column was washed with 100 ml lysis buffer to remove all non-His-tagged proteins. GDGT–MAS was eluted from the column with 75 ml elution buffer (50 mM HEPES, pH 7.5, 300 mM KCl, 10% glycerol, 10 mM BME and 300 mM imidazole). The eluate was concentrated in a 30-kDa MWCO Amicon Ultra Centrifugal Filter. GDGT–MAS was buffer exchanged into storage buffer (50 mM HEPES, pH 7.5, 300 mM KCl, 20% glycerol and 1 mM DTT) via a PD-10 desalting column. The resulting protein mixture was further purified by size-exclusion chromatography on a HiPrep 26/60 S200 column with an isocratic method using S200 buffer (50 mM HEPES, pH 7.5, 300 mM KCl, 10% glycerol and 10 mM DTT) as mobile phase. Fractions indicative of monomeric GDGT–MAS were pooled, concentrated and then buffer exchanged into storage buffer before flash-freezing and storage in liquid nitrogen. The resulting protein is referred to as ‘as-isolated GDGT–MAS’.

### *M. acetivorans* lipid extractions

*M. acetivorans* cell lysate was produced in William Metcalf’s Laboratory at the University of Illinois at Urbana-Champaign. *M. acetivorans* C2A strains were grown in HS medium with 50 mM trimethylamine^47^. Total archaeal lipid extraction was performed in a single-phase by resuspension of the lyophilized lysate (dry weight of approximately 25 mg) in 1.0 ml 2-propanol (IPA):water:EtOAc (30:10:60, v:v:v)^48^. The mixture was vortexed for 9 min, sonicated for 15 min, then centrifugation for 5 min at 15,000*g* at 4 °C to separate organic and aqueous layers. The organic upper phase was collected. The above extraction procedure was performed two more times on the aqueous phase. The combined organic phases were evaporated to dryness by nitrogen gas. For liquid chromatography–MS analysis, the dried lipid extract was resuspended in 100 µl IPA:acetonitrile (ACN):water (45:35:20, v:v:v). For GDGT–MAS archaeal lipid exchange and assays, the dried lipid extract was resuspended in 100 µl of 50 mM HEPES, pH 7.5, by mechanically stirring the solution at 50 °C for 2 h.

### GDGT–MAS archaeal lipid exchange

The bacterial phospholipids pulled down during purification of GDGT–MAS were exchanged with archaeal lipids by incubation of a 1.0 ml solution containing 20 µM Tes, 5′-dAH at 2.5 mM, methionine at 2.5 mM and 200 µl *M. acetivorans* lipid extract or 50 µM AG in storage buffer at 40 °C for 30 min. Following incubation, the solution was exchanged into the storage buffer via a PD-10 desalting column to remove excess lipids. Finally, GDGT–MAS was concentrated in a 30-kDa MWCO Amicon Ultra Centrifugal Filter. The resulting protein is referred to as ‘*M. acetivorans* lipid-exchanged GDGT–MAS’ when *M. acetivorans* lipid extract was used for the lipid exchange and ‘AG lipid-exchanged GDGT–MAS’ when synthesized AG lipid was used.

### Construction of GDGT–MAS variants

GDGT–MAS variants were generated by site-directed mutagenesis PCR using pMj0619 as a template and the primers described in Supplementary Table 1. After amplification, PCR products were digested with DpnI to remove parental plasmid. Sequence-verified constructs were used to transform the *E. coli* BL21(DE3) strain with pDB1282 and pBAD42-BtuCEDFB as described above for overexpression.

### Synthesis of substrate

Synthesis of the GDGT–MAS substrate AG was carried out as previously described^32^. Characterization matched that previously reported.

### GDGT–MAS and GDGT–MAS variants activity assays

Activity assays were carried out in triplicate and, unless otherwise stated, contained 2.5 µM GDGT–MAS or GDGT–MAS variant, 10 µM AG, 300 µM SAM, 1 mM TiCitrate, 200 mM KCl and 10 µM d-methionine-methyl-d_3_ in 75 mM HEPES, pH 7.5. At each time point, two aliquots were taken from the reaction. For lipid analysis, an aliquot of the reaction was quenched by a fivefold dilution in IPA:ACN (56.3:43.7 v/v) containing 1 µM phosphatidylglycerol 12:0. For analysis of 5′-dAH, an aliquot of the reaction was quenched by a twofold dilution in 150 mM sulfuric acid.

For quantification of 5′-dAH, reaction aliquots that were quenched in 150 mM sulfuric acid were centrifuged at 13,100*g* for 15 min at 4 °C to remove any precipitate. The supernatant was then injected onto an Agilent Technologies 1290 Infinity II series UHPLC system coupled to a 6470 QQQ Agilent Jet Stream electrospray-ionization mass spectrometer. Analytes were chromatographically separated on an Agilent Zorbax Extend-C18 RRHD column (2.1 mm × 50 mm, 1.8-µm particle size) at 32.5 °C that was equilibrated in 95% solvent A (0.1% formic acid, pH 2.6) and 5% solvent B (acetonitrile). Throughout the duration of a single injection, the following gradient was applied: from 0 to 0.5 min, solvent B was held at 5%; from 0.5 to 5 min, solvent B increased from 5% to 35%; and from 5 to 6.5 min, solvent B increased to 90%. Analytes were detected in positive mode using a multiple-reaction method. A standard curve of 5′-dAH (500 nM to 50 µM) with 5 µM d-methionine-methyl-d_3_ (internal standard) was prepared for quantification of 5′-dAH using the Agilent MassHunter Quantitative Analysis 10.1 software.

For lipid analysis, reaction aliquots that were quenched in IPA:ACN (56.3:43.7 v/v) containing 1 µM phosphatidylglycerol 12:0 were centrifuged at 13,100*g* for 15 min at 4 °C to remove any precipitate. The supernatant was then injected onto a Thermo Scientific Vanquish UHPLC system coupled to a Thermo Scientific Q Exactive HF-X mass spectrometer with an H-ESI ion source. Lipids were chromatographically separated on an Agilent Zorbax Extend-C18 column (4.6 mm × 50 mm, 1.8-µm particle size) at 45 °C that was equilibrated in 60% solvent A (60:40 water:ACN with 10 mM ammonium formate and 0.1% formic acid) and 40% solvent B (90:10 isopropanol:ACN with 10 mM ammonium formate and 0.1% formic acid). Throughout the duration of a single injection, the following gradient was applied: from 0 to 2 min, solvent B increased to 75%; from 2 to 11 min, solvent B increased to 85%; and from 11 to 17 min, solvent B increased to 99%. Analytes were detected in negative mode using a full-scan method with an H-ESI capillary temperature of 320 °C. From 0 to 12 min, the full-scan MS was collected with an in-source CID of 50.0 eV, a resolution of 120,00, an AGC target of 3 × 10^6^, and a scan range set to *m*/*z* 400–2,500. From 12 to 24 min, the full-scan MS was collected with an in-source CID of 30.0 eV, a resolution of 60,00, an AGC target of 1 × 10^6^, and a scan range set to *m*/*z* 1,200–2,000. The response ratio of analytes was determined relative to the 1 µM phosphatidylglycerol 12:0 internal standard.

### GDGT–MAS structure determination by X-ray crystallography

#### General crystallographic methods

X-ray diffraction datasets were collected at the General Medical Sciences and Cancer Institutes Collaborative Access Team (GM/CA-CAT) at the Advanced Photon Source, Argonne National Laboratory and the Berkeley Center for Structural Biology (BCSB) beamlines at the Advanced Light Source at Lawrence Berkeley National Laboratory. All datasets were processed using the HKL2000 or HKL3000 package, and structures were determined by single anomalous dispersion phasing using Autosol/HySS or by molecular replacement using the program PHASER^49–52^. Model building and refinement were performed with Coot and phenix.refine, respectively^49,53^. Ligand geometric restraints were obtained from the Grade Web Server (Global Phasing)^54^. Structures were validated and analysed for Ramachandran outliers with the Molprobity server^55^. Figures were prepared using PyMOL^56^. Active site cavity mapping was prepared using Hollow^57^.

#### Crystallization and structure determination of GDGT–MAS with bound LPP–5′-dAH–Met

Brown, plate-shaped as-isolated GDGT–MAS crystals were generated via the hanging drop vapour diffusion method at room temperature by mixing 1 µl of a solution of GDGT–MAS in storage buffer (5 mg ml^−1^) with 1 µl of the well solution (0.3 M sodium thiocyanate, 15% (w/v) PEG 3350, 2.5 mM 5'-dAH and 2.5 mM methionine). Crystals were prepared for data collection by mounting on rayon loops followed by soaking in cryoprotectant solution (perfluoropolyether oil (Hampton Research)) and flash-freezing in liquid nitrogen.

Diffraction datasets for single-wavelength anomalous diffraction phasing were collected at the Fe *K-*edge X-ray absorption peak (1.73818 Å). A native dataset was collected on a separate crystal. Initial phasing attempts in phenix.autosol revealed that GDGT–MAS probably contained four distinct iron-containing metallocofactors^49^. Enhanced phase information was obtained by modelling three of these heavy-atom sites as [Fe_4_S_4_] clusters^58^. Subsequent phasing in phenix.autosol with 13 iron sites yielded high-quality electron density maps suitable for model building^49^. The enhanced overall figure-of-merit was 0.36 and the Bayes-CC was 44.7 (ref. ^49^). Phenix.autobuild was used to generate an initial model of 366 residues out of 506 with an Rwork/Rfree of 0.30/0.37. The resulting model was then manually adjusted in Coot and refined in Phenix^49,53^. This model was then used as the search model in phasing the native dataset by molecular replacement in Phenix. The final model consists of residues −2 to 0 (residues on the expression tag), 1–375, 382–501, three [Fe_4_S_4_] clusters, one Fe(II) ion, one 5'-dAH, one Met and two molecules of phosphatidic acid (LPP). Ramachandran analysis shows that 98.38% of residues are in favoured regions with the remaining 1.62% in allowed regions. Data collection and refinement statistics are provided in Extended Data Table 1.

#### Crystallization and structure determination of GDGT–MAS with bound L1P–L4P–5′-dAH–Met

Brown, plate-shaped crystals of lipid-exchanged GDGT–MAS were generated via a hanging drop vapour diffusion method at room temperature by mixing 1 µl of a solution of GDGT–MAS (5 mg ml^−1^) with 1 µl of the well solution (0.1 M MES, pH 6.5, 20% (w/v) PEG 300, 2.5 mM 5'-dAH and 2.5 mM methionine). Crystals were prepared for data collection by mounting on rayon loops followed by soaking in cryoprotectant solution (perfluoropolyether oil (Hampton Research)) and flash-freezing in liquid nitrogen.

The structure was, by molecular replacement, using the coordinates of the GDGT–MAS–LPP–5′-dAH–Met complex as the search model^50^. Manual model building and refinement were performed in Coot and Phenix, respectively^49,53^. Unmodelled electron density in the active site was assigned as two archaeal lipid molecules: L1P and AG. The final model consists of residues −1 to 0 (residues on the expression tag), 1–375, 380–397, 402–499, three [Fe_4_S_4_] clusters, one Fe(II) ion, one 5'-dAH molecule, one Met molecule, one L1P and one AG.

### Generation of a sequence similarity network

The Enzyme Function Initiative enzyme similarity tool (EFI-EST) (https://efi.igb.illinois.edu) was used to perform an all-by-all BLAST analysis of the InterPro family IPR034474 (current name ‘Methyltransferase_Class_D’ with 5,224 sequences) to create an initial sequence similarity network with an alignment score threshold of 75 (refs. ^59–61^). To eliminate protein fragments, the EFI fragment option was applied during the creation of the sequence similarity network to exclude UniProt-defined protein fragments. All networks were visualized and edited in Cytoscape^62^. The final sequence similarity network (Supplementary Fig. 5) contains 2,525 sequences represented as individual nodes with an alignment score of 112.

### AlphaFold model of GDGT–MAS

The AlphaFold model of GDGT–MAS from *M. jannaschii* can be accessed from UniProt accession number Q58036 (refs. ^63,64^).

### Native protein MS

Native protein MS was performed with heated electrospray ionization (HESI) (both positive and negative mode) either by direct infusion or via size-exclusion chromatography. In both cases, data were collected on a Thermo QExactive HF-X operating in high-mass range mode. In the former, the protein sample was buffer exchanged into anaerobic buffer (200 mM ammonium acetate in anaerobic high-performance liquid chromatography grade water) via multiple centrifuge cycles using a 30-kDa cut-off filter. The anaerobic sample was placed into a syringe equipped with a closed PEEK tubing, removed from the anaerobic chamber and connected to the syringe drive with a flow of 5 µl min^−1^. The closed line was opened and quickly connected to tubing from the ultra-performance liquid chromatography pump via a Tee, which was connected to the HESI source. The line from the ultra-performance liquid chromatography carried 50 mM N_2_-purged ammonium acetate at a flow rate of 50 µl min^−1^ (total flow into HESI source of 55 µl min−^1^). For size-exclusion chromatography–MS, a Yarra 1.8-µm size-exclusion chromatography X150 column was used with a flow rate of 20 µl min^−1^ of 50 mM N_2_-purged ammonium acetate.

Native protein mass spectra were collected as described below. Full-scan spectra were collected using Thermo Xcalibur version 4.2.47 over various time periods under full MS mode. Ligand ejection spectra (inset plots) were collected under AIF (*m*/*z* of 2,500–5,500) with the scan range set to *m*/*z* 300–1,000. Data were reviewed with Thermo Scientific FreeStyle 1.8 SP2; mass spectra were deconvoluted with Thermo Scientific BioPharma Finder 4.1. Specific data collection parameters for each figure are described in Supplementary Table 2.

### Reporting summary

Further information on research design is available in the Nature Research Reporting Summary linked to this article.

## Online content

Any methods, additional references, Nature Research reporting summaries, source data, extended data, supplementary information, acknowledgements, peer review information; details of author contributions and competing interests; and statements of data and code availability are available at 10.1038/s41586-022-05120-2.

### Supplementary information

Supplementary Information This file contains Supplementary Figs. 1–6 and Supplementary Tables 1 and 2.

Reporting Summary

## Figures and Tables

**Extended Data Table 1:**
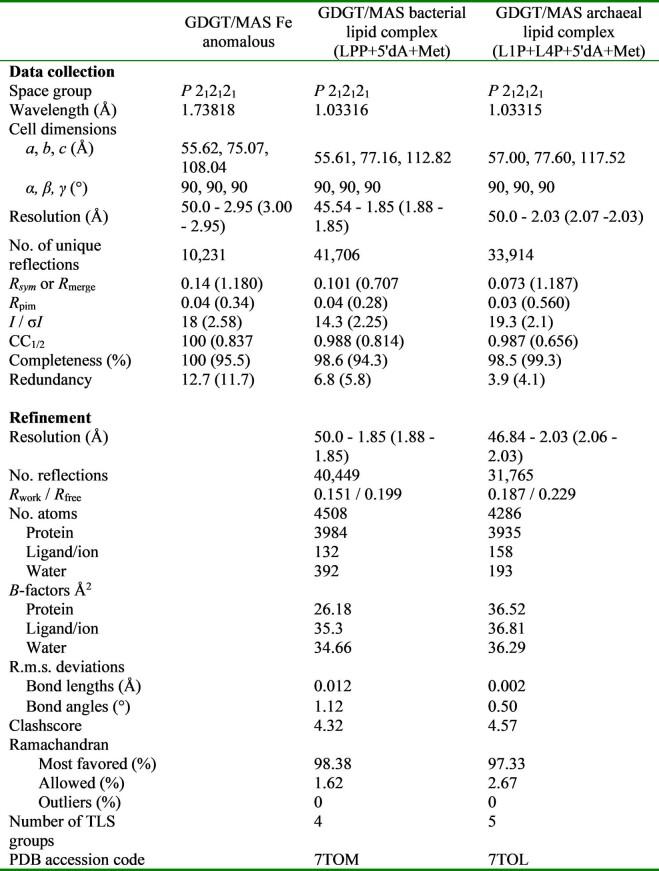
X-ray crystallographic data collection and refinement statistics

Values in parentheses represent the highest-resolution shell. All structures result from a single crystal.

## Data Availability

Atomic coordinates and structure factors for the reported crystal structures in this work have been deposited to the Protein Data Bank (PDB) under accession numbers 7TOL (archaeal lipid substrate + 5′-dAH + Met) and 7TOM (bacterial lipid substrate analogue + 5′-dAH + Met). The EFI-EST (https://efi.igb.illinois.edu) was used to perform an all-by-all BLAST analysis of the InterPro family IPR034474. Structures that were discussed but not reported in this work can be found at the following accession numbers: AlphaFold model of GDGT–MAS (UniProt accession number Q58036), GGR from *S. acidocaldarius* (PDB 4OPC) and CFAS from *E. coli* (PDB 6BQC).
