# Supplementary material for: Discovery, structure and mechanism of a tetraether lipid synthase
Source: Nature. 2022 Jul 26;609(7925):197–203. doi: 10.1038/s41586-022-05120-2 (PMC9433317; doi:10.1038/s41586-022-05120-2)
Supplement: Supplementary file 1 — This file contains Supplementary Figs. 1–6 and Supplementary Tables 1 and 2. [file 41586_2022_5120_MOESM1_ESM.pdf]

nature portfolio

https://doi.org/10.1038/s41586-022-05120-2

Supplementary information

Discovery, structure and mechanism of a tetraether lipid synthase

In the format provided by the authors and unedited

Supplementary Information for:

Discovery, structure, and mechanism of a tetraether lipid synthase

Authors: Cody T. Lloyd[1], David F. Iwig[2], Bo Wang[2], Matteo Cossu[4], William W. Metcalf[4,5], Amie K. Boal[1,2†], and Squire J. Booker[1,2,3†]

Affiliations: [1]Department of Biochemistry and Molecular Biology, Pennsylvania State University, University Park, PA; [2]The Howard Hughes Medical Institute, Pennsylvania State University, University. Park, PA, USA; [3]Department of Chemistry, Pennsylvania State University, University Park, PA, USA; [4]Department of Microbiology, University of Illinois Urbana–Champaign, Urbana, IL, USA; [5]Institute for Genomic Biology, University of Illinois Urbana– Champaign, Urbana, IL, USA

Figures S1-S6

Table S1-S2

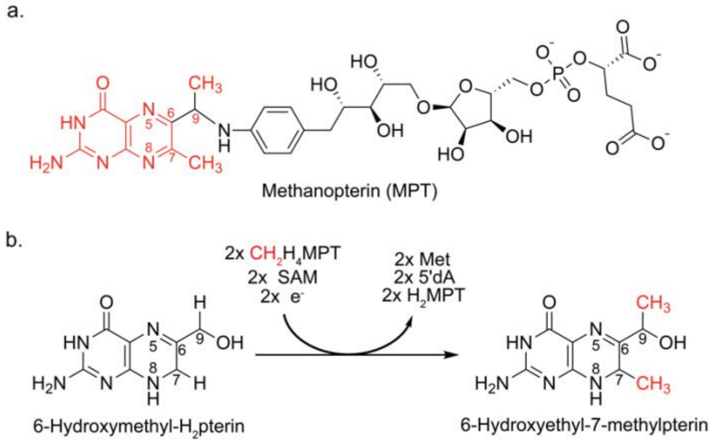

Supplementary Figure 1. Initial annotation of MJ0619 and the class D RS methylase subclass. GDGT/MAS was previously annotated as the pioneer enzyme—referred to as MJ0619—for the class D RSMT subclass and was proposed to methylate C7 and C9 on a pterin-like substrate during the biosynthesis of the methanopterin (MPT) cofactor. **a**, Chemical structure of methanopterin (MPT). The C7 and C9 methyl groups and the pterin moiety are in red. **b**, Proposed MJ0619 reaction, wherein each methylation requires SAM, one reducing equivalent, and a methyl donor, which was suggested to be methylenetetrahydromethanopterin (CH_2H_4MPT). Moreover, 6-hydroxymethyl-H_2pterin, a precursor in MPT biosynthesis, was hypothesized to be the substrate. Thus 6-hydroxyethyl-7-methylpterin would be the product. The proposed appended methyl groups are colored red.

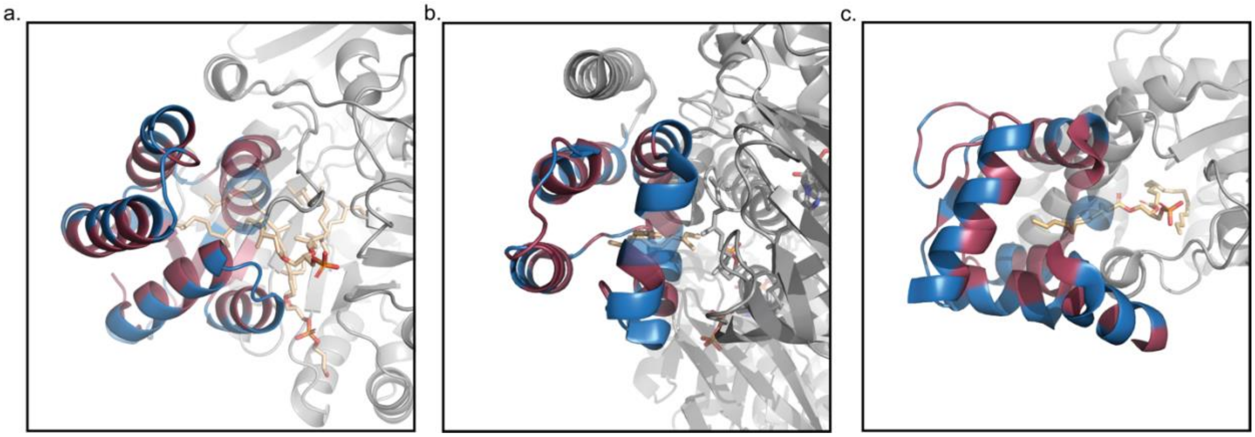

Supplementary Figure 2. Amphipathic alpha helices observed in known lipid modifying enzymes. a, GDGT/MAS **b,** Geranylgeranyl reductase (GGR) from *Sulfolobus acidocaldarius* (PDB 4OPC) **c,** cyclopropane fatty acid synthase from *E. coli* (6BQC). **a-c.** The amphipathic alpha-helical lipid-binding domains are colored blue for hydrophilic residues and maroon for hydrophobic residues. The alkyl lipid chains are colored wheat. In all structures, the hydrophobic residues of the lipid-binding domains are oriented towards the interior of the protein.

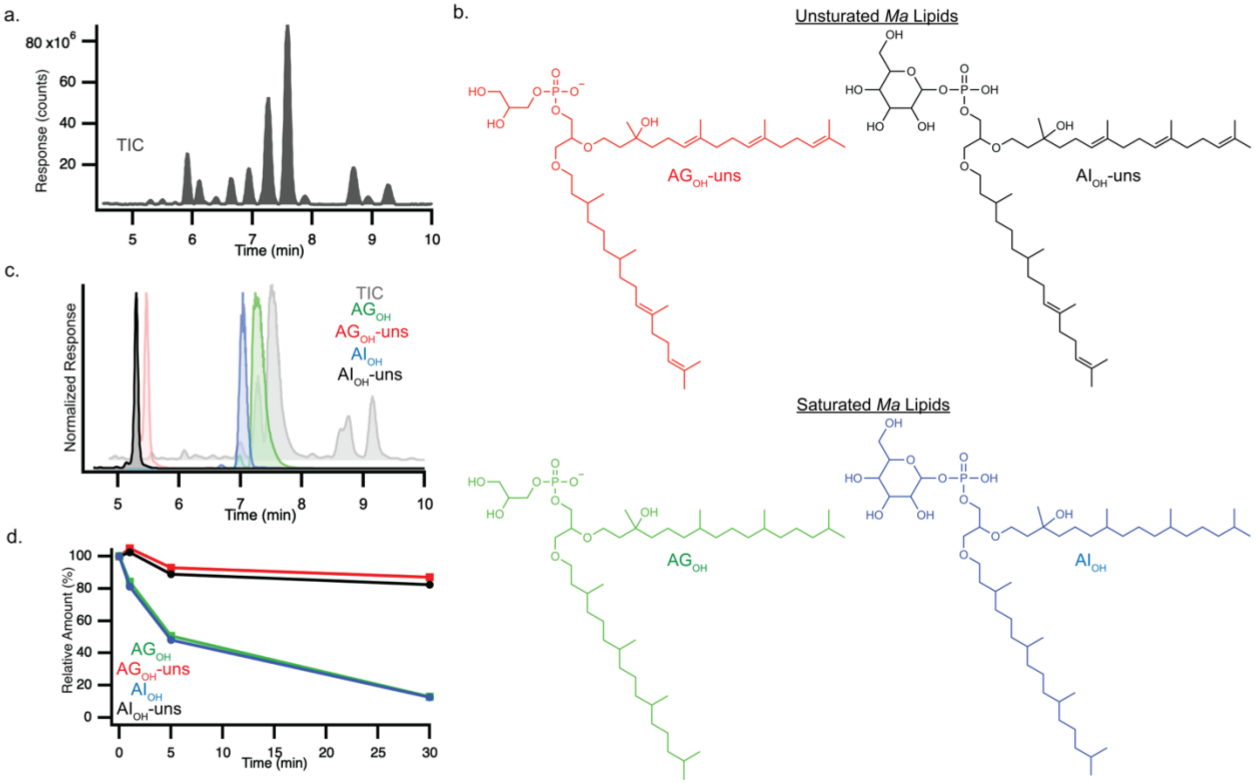

Supplementary Figure 3. *M. acetivorans* lipid extraction and GDGT/MAS assays with extracted *Ma* lipids. a. Total-ion chromatogram (TIC, from 4.5 – 10 min) of lipids extracted from *Ma* cell lysate. **b**. Structures of unsaturated and saturated *Ma* lipids monitored throughout the GDGT/MAS reaction. The highest degree of unsaturation detected in *Ma* lipid extract is five degrees of unsaturation observed in AG_{OH}-uns and AI_{OH}-uns. However, it is unclear where the unsaturations are on the carbon chain. The depicted location within this figure is one possibility. **c.** Extracted-ion chromatograms of saturated and unsaturated *Ma* lipids [3-hydoxyarchaetidylglycerol (AG_{OH}, green trace), unsaturated 3-hydoxyarchaetidylglycerol (AG_{OH}-uns, red trace), 3-hydoxyarchaetidylinositol (AG_{OH}, blue trace), and unsaturated 3-hydoxyarchaetidylinositol (AG_{OH}-uns, black trace)] overlayed with the TIC. **d.** Time-dependent monitoring of *Ma* lipids during a GDGT/MAS activity assay with *Ma* lipid extract as the substrate. These results suggest that GDGT/MAS solely catalyzes the modification of fully saturated (i.e., phytanyl chains) lipids. Trace coloring is identical to panel c.

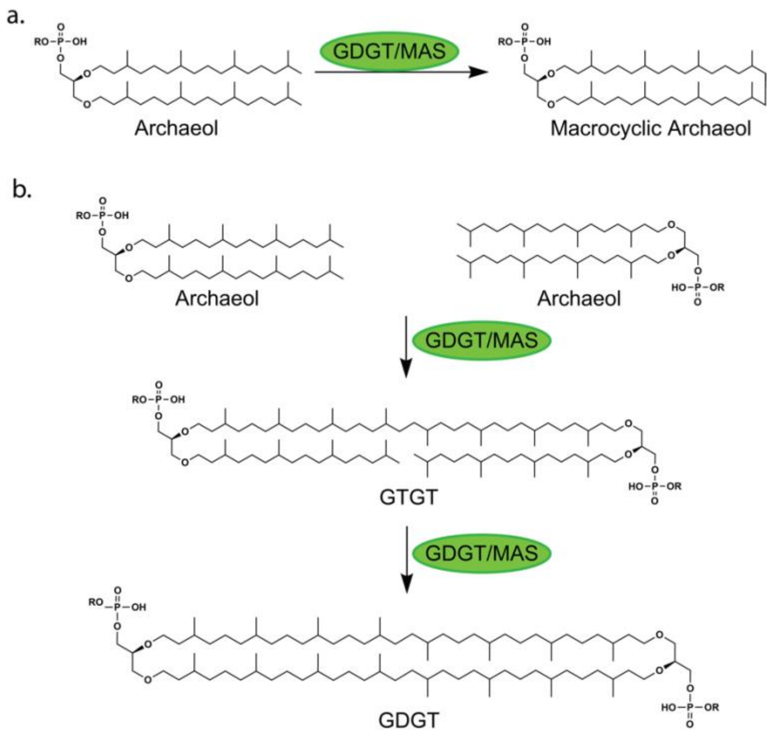

Supplementary Figure 4. GDGT/MAS catalyzes formation of the biphytanyl chain during synthesis of (a.) archaeol diether macrocycle and (b.) GDGT.

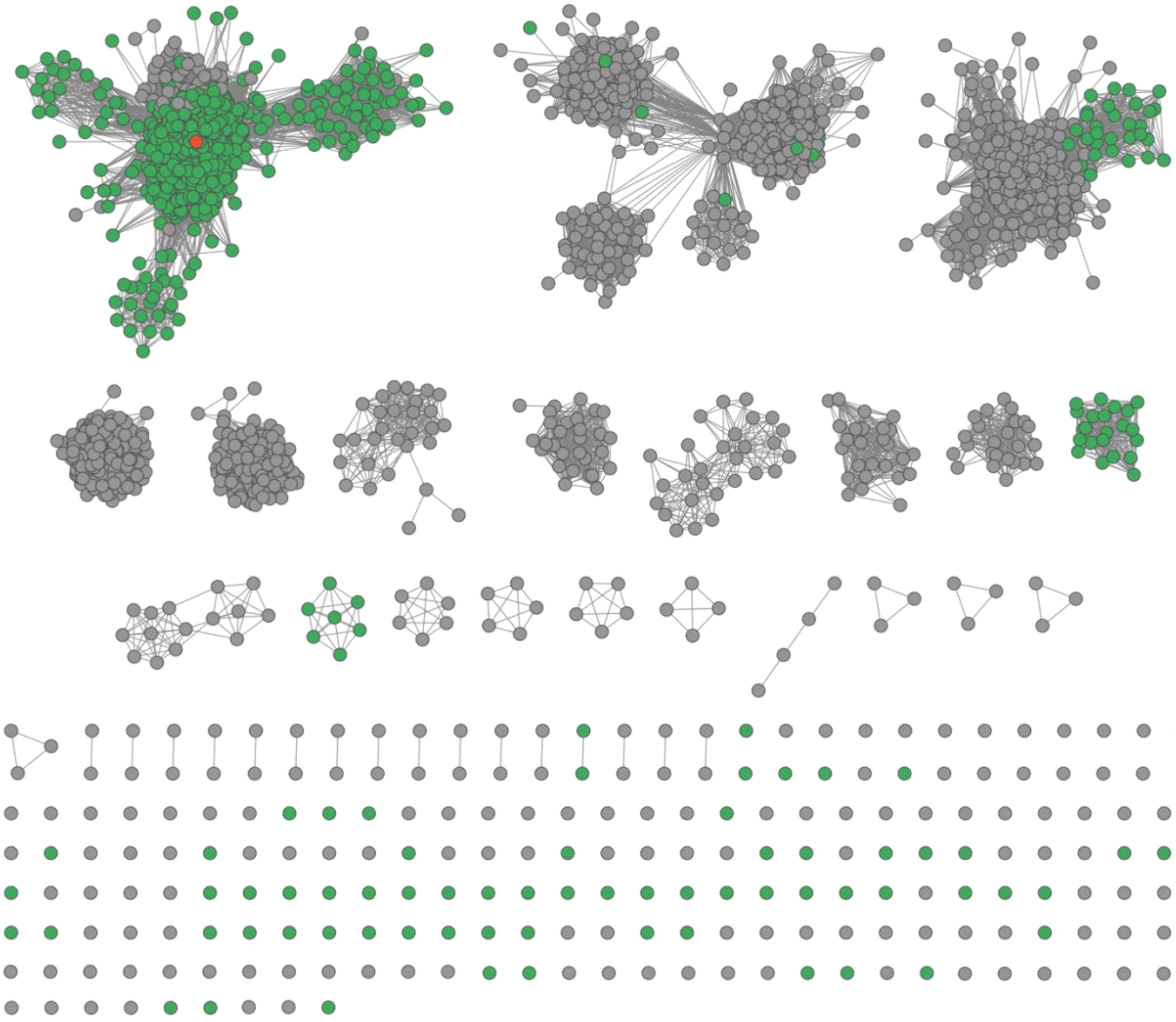

Supplementary Figure 5. Sequence similarity network of InterPro protein family (IPR034474) associated with GDGT/MAS. SSN of IPR034474 UniRef90 (2,525 sequences) was generated with an alignment score of 112. Each node represents a single sequence. Sequence clusters and nodes are colored green for Achaea and gray for Bacteria. The red node represents *Mj* GDGT/MAS. This SSN shows that GDGT/MAS homologs exists in archaeal and bacterial organisms.

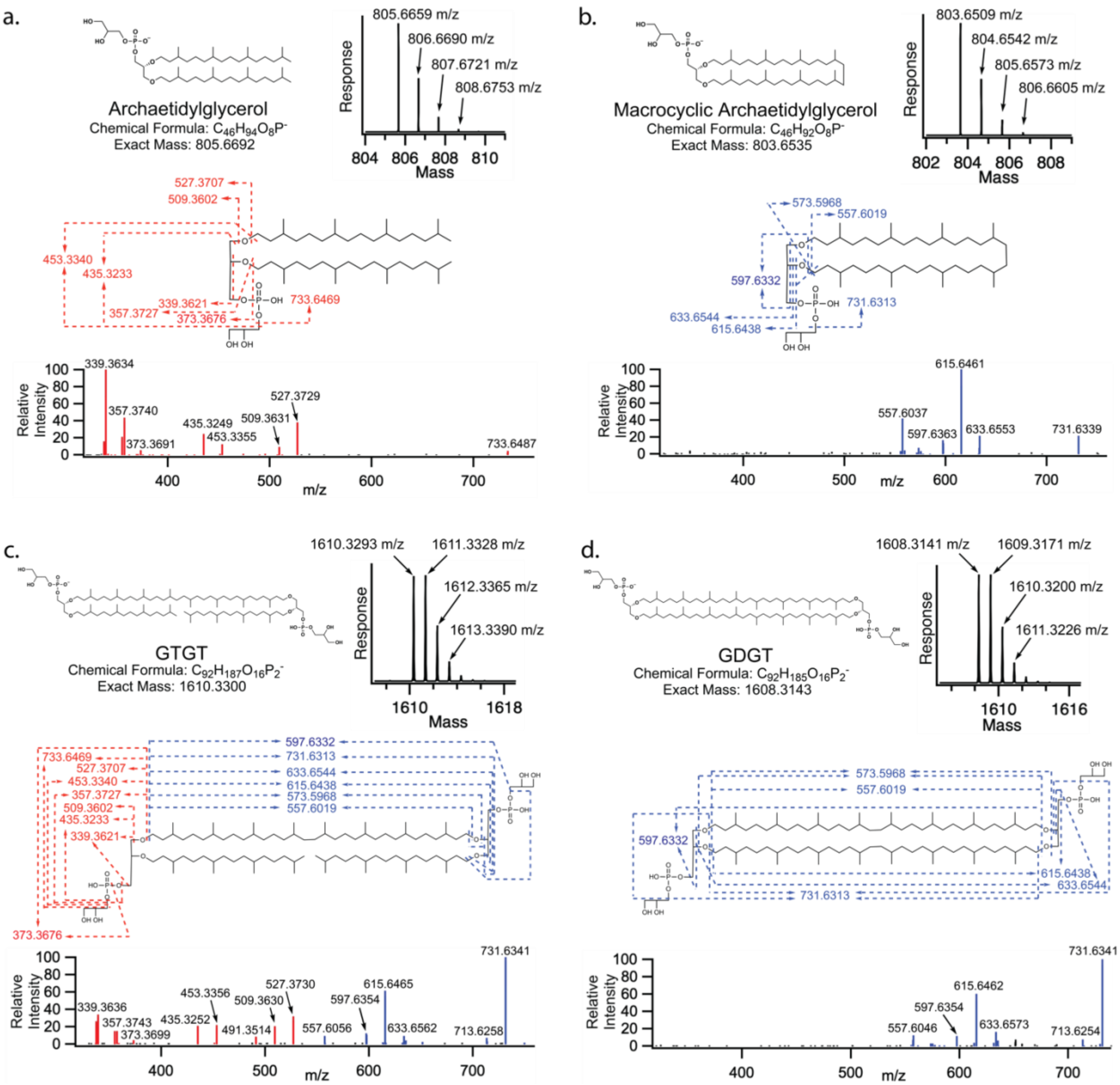

Supplementary Figure 6. Structural characterization of archaetidylglycerol (AG), macrocyclic AG, GTGT, and GDGT by high-resolution MS and tandem MS/MS. Panels **a-d** have an identical layout. **a,** Structural characterization of AG with the structure, chemical formula, and exact mass in the upper left corner of the panel. The mass spectrum in the upper right corner shows the *m/z* values observed for AG that result from natural abundance ^{13}C. ^{13}C has a natural abundance of approximately 1.1 percent. Therefore, a molecule with forty-six carbons will result in a mass spectrum with a relative abundance of forty-six percent for the *m/z* value of molecules that contain one ^{13}C isotope. The relative responses observed for the natural abundance ^{12}C isotope (*m/z* 805.6659) compared to the ^{13}C isotope (*m/z* values 806.6690, 807.6721, and 808.6753) corroborates that the chemical formula contains forty-six carbon atoms. The product ions resulting from tandem MS/MS fragmentation of AG are shown at the bottom of the panel with dashed lines identifying the location of the fragmented bond and the resulting theoretical *m/z*. All observed *m/z* values from tandem MS/MS are displayed on the mass spectrum. In the tandem MS/MS spectra, dashed lines and product ions colored red indicate the presence of a phytanyl chain, while blue coloring indicates the presence of a biphytanyl chain. **b,** Macrocyclic archaetidylglycerol **c,** Glycerol trialkyl glycerol tetraether (GTGT) **d,** Glycerol dibiphytanyl glycerol tetraether (GDGT).

Primer Name	Primer Sequence (5′ to 3′)
GDGT/MAS_Y459F_Forward	GTTGCTGCATTCATTTTGCAACACCGGATG
GDGT/MAS_Y459F_Reverse	CATCCGGTGTTGCAAAATGAATGCAGCA
GDGT/MAS_Y459L_Forward	GTTGCTGCATTCATCTGGCAACACCGGATG
GDGT/MAS_Y459L_Reverse	CATCCGGTGTTGCCAGATGAATGCAGCAAC
GDGT/MAS_M439A_Forward	GATTAGCTGCGCGCACTTTATGGATGCC
GDGT/MAS_M439A_Reverse	GGCATCCATAAAGTGCGCGCAGCTAATC

Table S1. Primers used for site-directed mutagenesis of GDGT/MAS

	Figure 6a	Figure 6a inset	Figure 6b	Figure 6b inset	Figure 6c	Figure 6c inset
Mode	ESI(-) SEC/MS	ESI(-) Direct	ESI(-) Direct	ESI(-) Direct	ESI(-) Direct	ESI(-) Direct
SID (eV)	110	100	100	110	50	115
Resolution	15,000	120,000	15,000	120,000	15,000	120,000
Microscans	8	1	8	1	10	1
AGC Target	1e6	1e6	1e6	1e6	1e6	1e6
Funnel RF	75	75	70	75	125	65
Cap. Temp.	290	290	290	290	300	320
HCD nCE (eV)	N/A	30	N/A	30	N/A	30

Table S2. Instrument parameters for native protein mass spectrometry for each mass spectra.
