# Supplementary material for: Discovery, structure and mechanism of a tetraether lipid synthase
Source: Nature. 2022 Jul 26;609(7925):197–203. doi: 10.1038/s41586-022-05120-2 (PMC9433317; doi:10.1038/s41586-022-05120-2)
Supplement: Supplementary file 2 — Reporting Summary [file 41586_2022_5120_MOESM2_ESM.pdf]

nature portfolio

Corresponding author(s): Squire J. Booker

Last updated by author(s): June 7, 2022

Reporting Summary

Nature Portfolio wishes to improve the reproducibility of the work that we publish. This form provides structure for consistency and transparency in reporting. For further information on Nature Portfolio policies, see our Editorial Policies and the Editorial Policy Checklist.

Statistics

For all statistical analyses, confirm that the following items are present in the figure legend, table legend, main text, or Methods section.

n/a	Confirmed	
☐	☒	The exact sample size (n) for each experimental group/condition, given as a discrete number and unit of measurement
☐	☒	A statement on whether measurements were taken from distinct samples or whether the same sample was measured repeatedly
☒	☐	The statistical test(s) used AND whether they are one- or two-sided<br>*Only common tests should be described solely by name; describe more complex techniques in the Methods section.*
☒	☐	A description of all covariates tested
☒	☐	A description of any assumptions or corrections, such as tests of normality and adjustment for multiple comparisons
☐	☒	A full description of the statistical parameters including central tendency (e.g. means) or other basic estimates (e.g. regression coefficient) AND variation (e.g. standard deviation) or associated estimates of uncertainty (e.g. confidence intervals)
☒	☐	For null hypothesis testing, the test statistic (e.g. F, t, r) with confidence intervals, effect sizes, degrees of freedom and P value noted<br>*Give P values as exact values whenever suitable.*
☒	☐	For Bayesian analysis, information on the choice of priors and Markov chain Monte Carlo settings
☒	☐	For hierarchical and complex designs, identification of the appropriate level for tests and full reporting of outcomes
☒	☐	Estimates of effect sizes (e.g. Cohen's d, Pearson's r), indicating how they were calculated

Our web collection on statistics for biologists contains articles on many of the points above.

Software and code

Policy information about availability of computer code

Data collection	All GDGT/MAS crystallographic datasets were processed using the HKL2000 or HKL3000 packages.The Enzyme Function Initiative enzyme similarity tool (EFI-EST) (https://efi.igb.illinois.edu) was used to perform an all-by-all BLAST analysis of the InterPro family IPR034474.
Data analysis	The Phenix suite (including phenix.refine, Autosol, PHASER, and Autobuild) v. 1.19.2-4158 was used for GDGT/MAS structural determination crystallography data refinement. Coot 0.9.6. was used for crystallography modeling. PyMOL 2.4.1 was used to make crystallography figures. HOLLOW v1.2 was used for visualization of protein cavities. Cytoscape 3.9.0 was used for analysis of the SSN. Liquid-chromatography mass-spectrometry data was analyzed using Thermo Xcalibur v. 4.2.47, Thermo Scientific FreeStyle v. 1.8 SP2, Thermo Scientific BioPharma Finder 4.1, and Agilent MassHunter Quantitative Analysis 10.1.

For manuscripts utilizing custom algorithms or software that are central to the research but not yet described in published literature, software must be made available to editors and reviewers. We strongly encourage code deposition in a community repository (e.g. GitHub). See the Nature Portfolio guidelines for submitting code & software for further information.

Data

Policy information about availability of data

All manuscripts must include a data availability statement. This statement should provide the following information, where applicable:

- Accession codes, unique identifiers, or web links for publicly available datasets
- A description of any restrictions on data availability
- For clinical datasets or third party data, please ensure that the statement adheres to our policy

Atomic coordinates and structure factors for the reported crystal structures in this work have been deposited to the Protein Data Bank (PDB) under the accession numbers 7TOL (GDGT/MAS archaeal lipid complex) and 7TOM (GDGT/MAS bacterial lipid complex). Structural data discussed, but not initially reported, in this work

can be found at the following accession numbers: AlphaFold model of GDGT/MAS (UniProt accesion number Q58036), Geranylgeranyl reductase from Sulfolobus acidocaldarius (PDB 4OPC), and cyclopropane fatty acid synthase from E. coli (PDB 6BQC).

Field-specific reporting

Please select the one below that is the best fit for your research. If you are not sure, read the appropriate sections before making your selection.

☒ Life sciences ☐ Behavioural & social sciences ☐ Ecological, evolutionary & environmental sciences

For a reference copy of the document with all sections, see nature.com/documents/nr-reporting-summary-flat.pdf

Life sciences study design

All studies must disclose on these points even when the disclosure is negative.

Sample size	Structures from individual protein crystals were solved by SAD phasing. Sample size calculation is not applicable to this study, because single protein crystals were solved. Assays were performed with a sample size of three (i.e., triplicate), which is the standard sample size for in vitro enzymology. Each assay sample was prepared and data was collected individually.
Data exclusions	Anomalous and native GDGT/MAS datasets were integrated and scaled using the HKL2000 and HKL3000 package, and no data exclusion was applied. Activity assays were performed in triplicate, and no data was excluded.
Replication	GDGT/MAS activity assays were performed in triplicate and noted. All attempts of replication were successful.
Randomization	Randomization is not applicable to this study, because single protein crystal structures were solved.
Blinding	No crystal structure of this enzyme exists and as such, no expectations were made on how it would appear. Thus, blinding was not necessary for this study.

Reporting for specific materials, systems and methods

We require information from authors about some types of materials, experimental systems and methods used in many studies. Here, indicate whether each material, system or method listed is relevant to your study. If you are not sure if a list item applies to your research, read the appropriate section before selecting a response.

Materials & experimental systems

n/a	Involved in the study	
☒	☐	Antibodies
☒	☐	Eukaryotic cell lines
☒	☐	Palaeontology and archaeology
☒	☐	Animals and other organisms
☒	☐	Human research participants
☒	☐	Clinical data
☒	☐	Dual use research of concern

Methods

n/a	Involved in the study	
☒	☐	ChIP-seq
☒	☐	Flow cytometry
☒	☐	MRI-based neuroimaging
